# Eight-Fold Interpenetrating Diamondoid Coordination Polymers for Sensing Volatile Organic Compounds and Metal Ions

**DOI:** 10.3390/polym13183018

**Published:** 2021-09-07

**Authors:** Venkatesan Lakshmanan, Yi-Ting Lai, Xiang-Kai Yang, Manivannan Govindaraj, Chia-Her Lin, Jhy-Der Chen

**Affiliations:** 1 Department of Chemistry, Chung-Yuan Christian University, Chung Li 32023, Taiwan; venkatesanflower95@gmail.com (V.L.); e0912583362@gmail.com (Y.-T.L.); xiangkaishulin@gmail.com (X.-K.Y.); manivannanjent@gmail.com (M.G.); 2 Department of Chemistry, National Taiwan Normal University, Taipei 11677, Taiwan

**Keywords:** coordination polymer, crystal structure analysis, entanglement, luminescence

## Abstract

Reactions of divalent metal salts with 4,4-oxybis(N-(pyridine-4-yl)-benzamide), **L**, and naphthalene-1,4-dicarboxylic acid (1,4-H_2_NDC) in various solvents gave [Zn(**L**)(1,4-NDC)·H_2_O]_n_, **1**, [Cd(**L**)(1,4-NDC)(H_2_O)·MeOH]_n_, **2**, and [Co(**L**)(1,4-NDC)(H_2_O)_0.5_·MeOH]_n_, **3**, which have been structurally characterized. Complexes **1**–**3** show eight-fold interpenetrating frameworks with the **dia** topology, which exhibit porosities substantiated by CO_2_ adsorption, whereas **1** and **2** manifest stability in aqueous environments and show high selectivity toward sensing of mesitylene molecules and Fe^3+^ ions with low detection limits and good reusability up to five cycles.

## 1. Introduction

Coordination polymers (CPs) have shown potential applications in luminescent sensing, gas storage and separation, drug delivery, catalysis, ion exchange and magnetism [1,2,3,4,5]. Metal ions are linked together by organic ligands to form extended frameworks, which are often porous and comprised into infinite arrays. In addition, polycarboxylates play a vital role in the organization of diverse CPs with differential dimensionalities, which show multiple potential coordination sites that are highly active to coordinate with metal centers. Linear and angular types of polycarboxylate ligands have been used extensively in the preparation of CPs in a mixed system [6,7]. Entanglement involving interpenetration, polycatenation and self-catenation is a very interesting phenomenon, which is also a major factor contributing to the diversities of CPs due to their aesthetic and intriguing topological interests [8]. Despite many such interesting CPs being reported, the manipulation of the structural diversity is still a challenge. 

Considerable effort has been invested into understanding the entanglement of CPs containing bis-pyridyl-bis-amide (bpba) ligands [9,10,11,12,13]. Two highly interpenetrated Zn(II) and Cd(II) CPs with eight- and nine-fold interpenetrating diamondoid modes, respectively, have been reported by using the long flexible *N*,*N*′-di(4-pyridyl)adipoamide ligand and the short rigid 1,4-benzenedicarboxylic acid [13]. Construction of a high-fold interpenetrating diamondoid framework with linear bidentate ligand may thus be ascribed to the length and flexibility of the spacer ligands and the geometrical arrangements of the metal centers that may modulate pore size and shape in a single network. While the entanglement may reduce the solvent accessible volume required for the molecules/metal ions adsorptions, the modulated pore size and shape may enhance the selectivity. Moreover, the tunable porosity of luminescent CPs has a great impact on the sensitivity and selectivity for the detection of volatile organic compounds (VOCs) and metal ions that are harmful to the environment and public health [14].

In this study, three CPs constructed from the angular ligand 4,4-oxybis(N-(pyridine-4-yl)-benzamide (**L**), 1,4-H_2_NDC and divalent metal salts, namely, [Zn(**L**)(1,4-NDC)·H_2_O]_n_, **1**, [Cd(**L**)(1,4-NDC)(H_2_O)·MeOH]_n_, **2**, and [Co(**L**)(1,4-NDC)(H_2_O)_0.5_·MeOH]_n_, **3**, are reported, which show eight-fold interpenetrating diamondoid networks. Complexes **1** and **2** exhibit different emission intensity enhancement for distinct VOCs, and show a high sensitivity toward detection of Fe^3+^ ion in aqueous systems. The synthesis, structural characterization and the detections of VOCs and metal ions of these CPs form the subject of this report. 

## 2. Materials and Methods

### 2.1. General Procedures

Elemental analyses of (C, H, N) were performed on a PE 2400 series II CHNS/O (PerkinElmer Instruments, Shelton, CT, USA) or an Elementar Vario EL III analyzer (Elementar Analysensysteme GmbH, Hanau, German). Infrared spectra were obtained from a JASCO FT/IR-460 Plus spectrometer with pressed KBr pellets (JASCO, Easton, MD, USA). Thermal gravimetric analyses (TGA) were carried out on an SII Nano Technology Inc. TG/DTA 6200 over the temperature range of 30 to 900 °C at a heating rate of 10 °C min^−1^ under N_2_ (SEIKO Instruments Inc., Chiba, Japan). UV–vis spectrum was performed on a UV-2450 spectrophotometer (Dongguan Hongcheng Optical Products Co., Dongguan, China). Emission spectra for the solid samples were determined with a Hitachi F-4500 fluorescence spectrophotometer (Hitachi, Tokyo, Japan). Gas sorption measurements were conducted using a Micromeritics ASAP 2020 system (Micromeritics Instruments Co., Norcross, GA, USA). Powder X-ray diffraction patterns were carried out with a Bruker D8-Focus Bragg-Brentano X-ray powder diffractometer equipped with a CuKα sealed tube (λ_α_ = 1.54178 Å) at 40 kV and 40 mA (Bruker Corporation, Karlsruhe, Germany).

### 2.2. Materials

The reagent Zn(OAc)_2_·2H_2_O was purchased from SHOWA Co. (Saitama, Japan), Cd(OAc)_2_·2H_2_O from Fisher Scientific Co. (Hampton, NY, USA), Co(OAc)_2_·4H_2_O from J. T. Baker Co. (Phillipsburg, NJ, USA), and naphthalene-1,4-dicarboxylic acid (1,4-H_2_NDC) from Alfa Aesar Co. (Ward Hill, MA, USA). The ligand 4,4′-oxybis(N-(pyridine-4-yl)-benzamide) (**L**) was prepared according to a published procedure with some modification [15].

### 2.3. Preparations

#### 2.3.1. [Zn(**L**)(1,4-NDC)·H_2_O]_n_, **1**

Zn(OAc)_2_·2H_2_O (0.022 g, 0.10 mmol), **L** (0.041 g, 0.10 mmol) and 1,4-H_2_NDC (0.022 g, 0.10 mmol) in 8 mL H_2_O and 2 mL methanol were sealed in a 23 mL Teflon-lined flask inside a stainless steel autoclave, which was heated at 120 °C for 2 days, and then the reaction system was cooled down at a rate of 2 °C per hour until the room temperature was reached. Colorless crystals were obtained, which were purified with methanol. Yield: 0.041 g (58%), using Zn(II) as the limited reagent. Anal. calcd. for C_36_H_26_N_4_O_8_Zn (MW = 707.98): C, 61.07; N, 7.91; H, 3.70%. Found: C, 61.22; N, 8.07; H, 4.01%. FT-IR (cm^−1^): 3237(m, N-H), 3168(m, N-H), 3070(w), 1686(s), 1596(s), 1512(s), 1490(s), 1429(m), 1332(m), 1236(m), 1210(m), 1170(s).

#### 2.3.2. [Cd(**L**)(1,4-NDC)(H_2_O)·MeOH]_n_, **2**

The procedures for **2** were similar to **1** except Cd(OAc)_2_·2H_2_O (0.013 g, 0.05 mmol), **L** (0.021 g, 0.05 mmol) and 1,4-H_2_NDC (0.011 g, 0.05 mmol) in 2 mL H_2_O and 8 mL methanol were used. Colorless crystals were obtained. Yield: 0.023 g (58%). Anal. calcd. for C_37_H_30_CdN_4_O_9_ (MW = 787.05): C, 56.34; N,7.11; H, 3.80%. Found: C, 55.69; N, 7.05; H, 3.44%. FT-IR (cm^−1^): 3168(m), 3068(m), 1664(s), 1595(s), 1552(s), 1494(s), 1426(s), 1364(s), 1334(s), 1289(m), 1235(s), 1211(m), 1169(s), 1100(m), 1016(m), 836(m), 760(m), 583(m).

#### 2.3.3. [Co(**L**)(1,4-NDC)(H_2_O)_0.5_·MeOH]_n_, **3**

Complex **3** was prepared according to the procedures for **1** except Co(OAc)_2_·4H_2_O (0.025 g, 0.10 mmol), **L** (0.041 g, 0.10 mmol) and 1,4-H_2_NDC (0.022 g, 0.10 mmol) were used. Purple crystals were obtained. Yield: 0.036 g (49%). Anal. calcd for C_37_H_29_CoN_4_O_8.5_ (MW = 724.57): C, 61.33; N, 7.73; H, 4.03%. Found: C, 60.50; N, 7.56; H, 3.90%. FT-IR (cm^−1^): 3230(m), 3158(m), 3069(m), 1683(m), 1595(s), 1510(s), 1494(s), 1427(s), 1363(s), 1330(s), 1274(m), 1235(s), 1208(m), 1170(m), 1098(m), 1022(m), 838(m), 759(w), 584(w). Figure S1 shows the FT-IR spectra of complexes **1**–**3**.

### 2.4. X-ray Crystallography

A Bruker AXS SMART APEX II CCD diffractometer, which was equipped with graphite-monochromated MoKα (λ_α_ = 0.71073 Å) radiation, was used to collect the diffraction data for complexes **1**–**3** [16], and the data were reduced by using the well-established computational procedures. The structure factors were treated with Lorentz and polarization corrections and an empirical absorption correction based on “multi-scan” [17]. The Direct or Patterson method was applied to locate the positions of some of the heavier atoms, and the remaining atoms were identified in several alternating difference Fourier maps and least-square refinements. Hydrogen atoms were added by using the HADD command in SHELXTL. The coordinated water molecule, O(8), of complex **2** is disordered such that two orientations of the oxygen atom can be found, and the occupancy of each orientation was set to be 0.5 and were refined isotropically. Moreover, the occupancy of O(8) of **3** was refined to be 0.5. Table 1 lists the basic information regarding the crystal parameters and structure refinement.

## 3. Results and Discussion

### 3.1. Crystal Structures of ***1**–**3***

**Figure 1:**
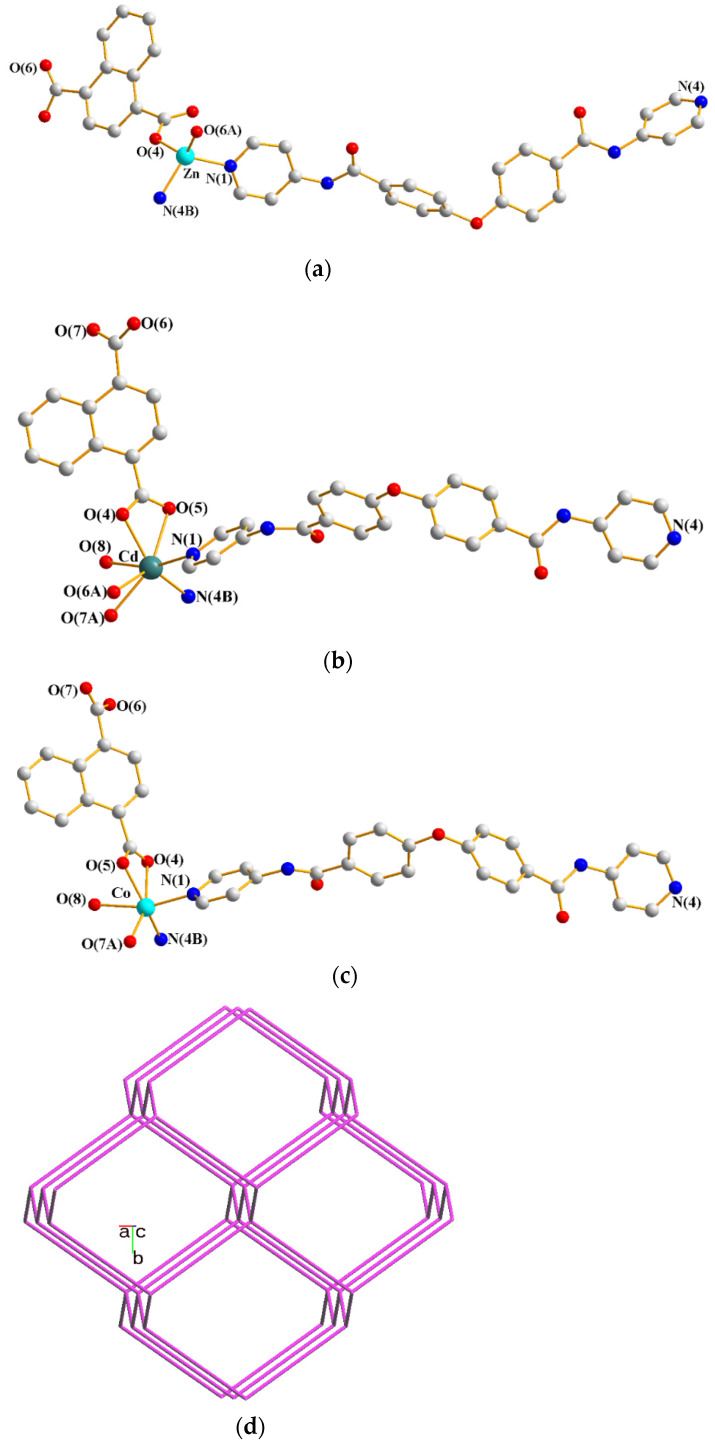

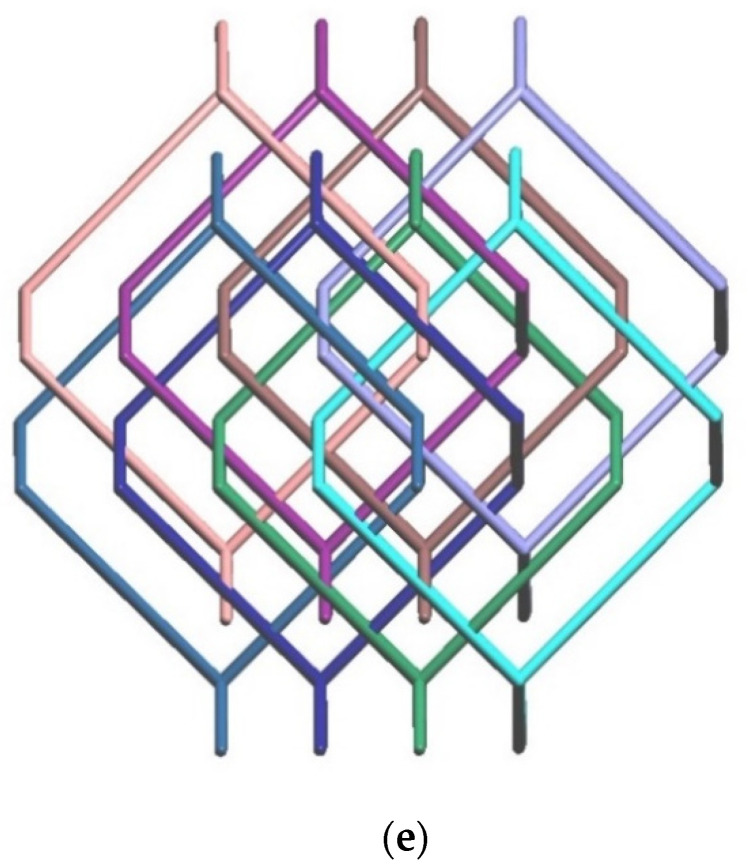
Coordination environments about the (**a**) Zn(II) ion in **1**. Symmetry transformations used to generate equivalent atoms: (A) x, −y + 5/2, z − 1/2; (B) x − 2, −y + 3/2, z − 1/2; (**b**) Cd(II) ion in **2**. Symmetry transformations used to generate equivalent atoms: (A) x + 1/2, −y + 3/2, z + 1/2; (B) x − 3/2, −y + 1/2, z + 1/2; and (**c**) Co(II) ion in **3**. Symmetry transformations used to generate equivalent atoms: (A) x − 3/2, −y + 1/2, z + 1/2; (B) x + 1/2, −y + 3/2, z + 1/2. (**d**) A drawing showing a 3D framework with the **dia** topology. (**e**) A schematic view of the eight-fold [4 + 4] interpenetration.

Complexes **1**–**3** are isostructural and their single crystals conform to the monoclinic system. While the crystal structure of **1** was solved in the space group *P*2_1_/*c*, those of **2** and **3** were in *P*2_1_/*n*. Their asymmetric units are mainly composed of one divalent metal ion [Zn(II), **1**; Cd(II), **2** and Co(II), **3**], one **L** and one 1,4-NDC^2−^ ligands, and there is also one cocrystallized water molecule in **1**, one coordinated water and one cocrystallized MeOH molecule in **2,** and a half coordinated water and one cocrystallized MeOH molecule in **3**, respectively. Figure 1a–c depict the drawings showing the coordination environments about the metal ions of complexes **1**–**3**. The Zn(II) ions of **1** is four-coordinated by two pyridyl nitrogen atoms [Zn-N = 2.024(3) Å and 2.037(3) Å] from two **L** ligands and two carboxylate oxygen atoms [Zn-O = 1.905(5) and 1.940(3) Å] from two 1,4-NDC^2−^ ligands, forming a distorted tetrahedral geometry, while the Cd(II) ion of **2** is seven-coordinated by two pyridyl nitrogen atoms [Cd-N = 2.272(5) and 2.297(5) Å] from two **L** ligands, four carboxylate oxygen atoms [Cd-O = 2.248(5)–2.577(4) Å] from two 1,4-NDC^2−^ ligands and one oxygen atom [Cd-O = 2.416(13) Å] of the coordinated water molecule, resulting in a distorted pentagonal bipyramidal geometry. As shown in the X-ray crystallographic section, the occupancy of the coordinated water of **3** was refined to be 0.5; six and five coordination are thus possible for the Co(II) ion. The six-coordinated Co(II) ion is supported by two pyridyl nitrogen atoms [Co-N = 2.069(5) and 2.088(6) Å] from two **L** ligands, three carboxylate oxygen atoms [Co-O = 1.913(10)–2.302(5) Å] from two 1,4-NDC^2−^ ligands and one oxygen atom [Co-O = 2.238(9) Å] of the coordinated water molecule, revealing a distorted octahedral geometry, while the removal of the O(8) atom results in a five-coordinated Co(II) ion and forms a distorted square pyramidal geometry. 

Considering the divalent metal ions as four-connection nodes and the ligands as linkers, the structures of **1**–**3** can be simplified as 3D nets with the (6^6^)-**dia** topology (Figure 1d) revealing the 8-fold [4 + 4] interpenetration (Figure 1e), determined using ToposPro [18]. Noticeably, the diamondoid Zn(II) and Cd(II) CPs constructed from the flexible *N*,*N*′-di(4-pyridyl)adipoamide and 1,4-benzenedicarboxylic acid show eight- and nine-fold interpenetrating modes, respectively [13], indicating the nature of the metal center alters the degree of interpenetration. In marked contrast, complexes **1** and **2** with the Zn(II) and Cd(II) ions, respectively, are both eight-fold interpenetrated, demonstrating that the degree of interpenetration of the diamondoid CPs in mixed systems is also subject to the changes of the shape and flexibility of the polycarboxylate and bpba ligands. 

### 3.2. Ligand Conformations and Bonding Modes

**Figure 2:**
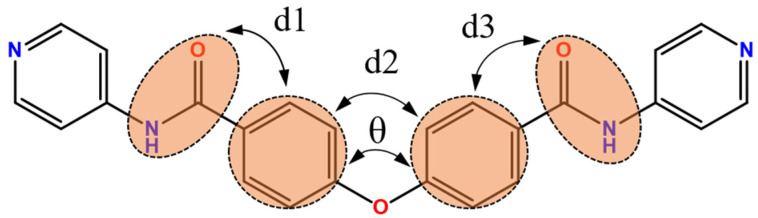
A drawing defining the C-O-C (θ) and dihedral angles (d) for **L**.

For the bpba ligand, the two C=O (or N-H) groups that are on the same and opposite directions define the *cis* and *trans* conformations, respectively [19]. Accordingly, all of the **L** ligands in **1**–**3** adopt the *cis* conformation. A schematic drawing defining the C-O-C (θ) angle and dihedral angle (d) is shown in Figure 2, and the angles were calculated and listed in Table 2. Clearly, although all of the **L** ligands in **1**–**3** adopt the same *cis* conformation, they differ in the dihedral angles, presumably due to the different metal identity. 

**Figure 3:**
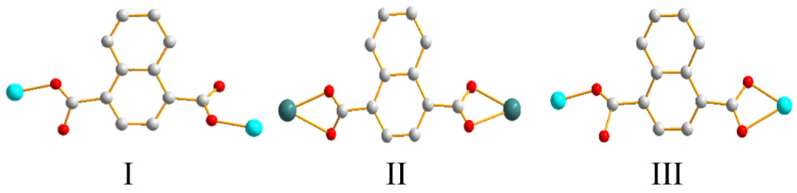
Various coordination modes of 1,4-NDC^2−^ ligands in **1**–**3**.

Moreover, the 1,4-NDC^2−^ ligand of **1** bridge two Zn(II) ions through two carboxylate oxygen atoms from two carboxylate groups to reveal the coordination mode I, while those of **2** and **3** chelate and bridge two metal ions through four carboxylate oxygen atoms, coordination mode II, and three carboxylate oxygen atoms, coordination mode III, respectively (Figure 3). Noticeably, while the Zn(II), Cd(II) and Co(II) metal centers of **1**–**3** adopt the distorted tetrahedral, pentagonal bipyramidal and octahedral (square pyramidal) geometries, respectively, giving rise to different coordination modes for the 1,4-NDC^2−^ ligands; their structural topologies are not subject to the metal identities.

### 3.3. PXRD Patterns and Thermal Analysis

As shown in Figures S2–S4, the experimental PXRD patterns of complexes **1**–**3** are in consistency with their corresponding simulated ones, demonstrating the purities of the bulk samples. On the other hand, their thermal gravimetric analysis (TGA) curves performed in a nitrogen atmosphere from 30 to 800 °C (Figure S5) display two-step weight losses involving removal of cocrystallized solvents and the coordinated ligands were found. From Table 3, it can be shown that the starting temperature for the removal of the organic ligands of **3**, 300 °C, is higher than those of **1**–**2**, probably indicating that the framework of **3** is stronger than those of **1**–**2** upon heating.

### 3.4. Chemical Stability

To estimate the chemical stability, the PXRD patterns of complexes **1**–**3** in water, as well as their dehydration and rehydration products, were measured. Complexes **1**–**3** were first immersed into water for seven days and then dried under vacuum. Figures S6–S8 show that only complexes **1** and **2** are stable in water. Moreover, dehydration (heated at 150 °C for 1 h) and rehydration (immersed into water for 1 h) of complexes **1** and **2** gave PXRD patterns comparable to the simulated ones, indicating the stability upon solvent removal and adsorption. The stabilities of **1** and **2** in acidic and basic solutions at 50 °C for 24 h were further investigated. As shown in Figures S9 and S10, the PXRD patterns of **1** in pH 2–12 and **2** in pH 4–10 are almost identical to the simulated ones, suggesting the better acid and base resistances of **1** than **2**.

### 3.5. Gas Sorption 

The low-pressure gas sorption properties at 273 K and 298 K were examined for complexes **1** and **2** and confirmed by CO_2_ gas absorption and desorption isotherms, resulting in a type II isotherm behavior [20,21]. As illustrated in Figures S11–S13, the experimental amount of adsorbed CO_2_ gas in the sample at 273 K is higher than that at 298 K. While desolvated samples of **1** and **2** adsorbed 1.40565 and 1.55795 mmol/g of CO_2_ at 273 K, they adsorbed 0.83070 and 1.05312 mmol/g at 298 K, respectively. The Brunauer–Emmett–Teller (BET) surface areas of **1** and **2** evaluated from the CO_2_ gas uptake are 411.3 and 302.4 m^2^ g^−1^ and the Langmuir surface areas are 482.6 and 330.5 m^2^ g^−1^ at 273 K, with total pore volumes of 0.05 and 0.06 cm^3^ g^−1^, respectively. The pore size distribution and sorption/desorption hysteresis are presumably related to very narrow pore sizes. Calculations using nonlocal density functional theory based on CO_2_ sorption data at 273 K suggest that the pore sizes for **1** and **2** are similar and are around 1.06 nm, respectively, indicating microporous materials. The PXRD patterns of the samples of **1** and **2** measured after the experiments match quite well with those of the simulated ones, indicating permanent porous features of **1** and **2** (Figure S13c,d). 

### 3.6. Photoluminescence Properties 

An ample number of studies have shown that CPs with d^10^ metal centers exhibit great sensing properties [22,23,24,25,26]. Therefore, the solid-state emission spectra of the water-stable **1** and **2**, as well as 1,4-H_2_NDC and **L** ligands, were investigated at room temperature and the results are shown in Figure S14 and Table 4. The emission band of **L** appears at 468 nm upon excitation at 376 nm, while 1,4-H_2_NDC exhibits an intense emission band at 480 nm upon two excitations at 280 and 370 nm, which may be attributed to intra-ligand π* → π or π* → *n* transition.

As-synthesized complexes **1** and **2** may contain water molecules in the pores, they were first activated by immersion into methanol for two days and heated at 80 °C for 1 h to give **1a** and **2a**, respectively. It can be seen (Figure S15) that complexes **1a** and **2a** show identical emission wavelengths to those of original **1** and **2**. Moreover, the PXRD patterns (Figure S16) of **1a** and **2a** match quite well with those of **1** and **2**, respectively, indicating no framework changes and suggesting ligand-based emissions with little contribution from the cocrystallized solvents. The different emission wavelengths in **1** and **2** are probably due to the distinction in the metal identity and the coordination mode of the 1,4-NDC^2−^ ligand. Zn(II) and Cd(II) atoms are more difficult to undergo oxidation and reduction reactions, due to their closed-shell electronic configurations; therefore, the emissions of complexes **1** and **2**, as well as **1a** and **2a**, are probably due to ligand-to-ligand charge transfer (LLCT), while metal-to-ligand charge transfer (MLCT) is also possible. 

### 3.7. Detection of Volatile Organic Compounds (VOCs) 

**Figure 4:**
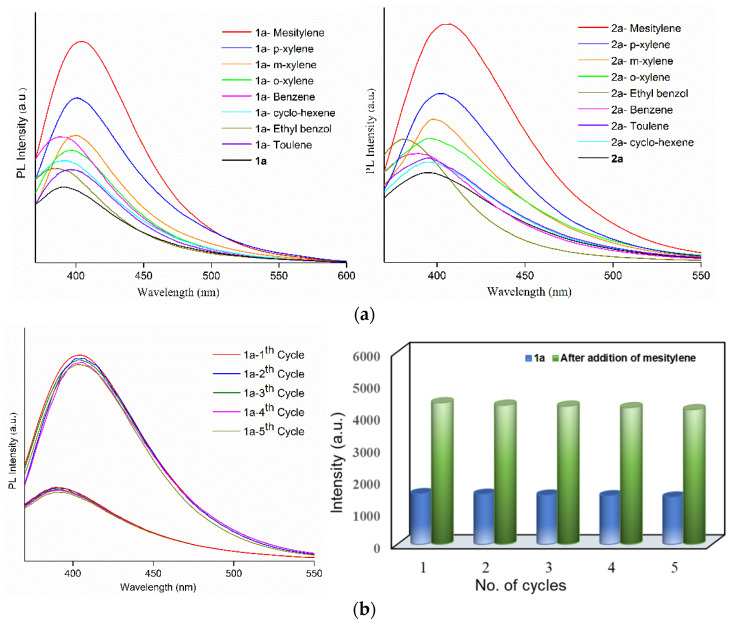

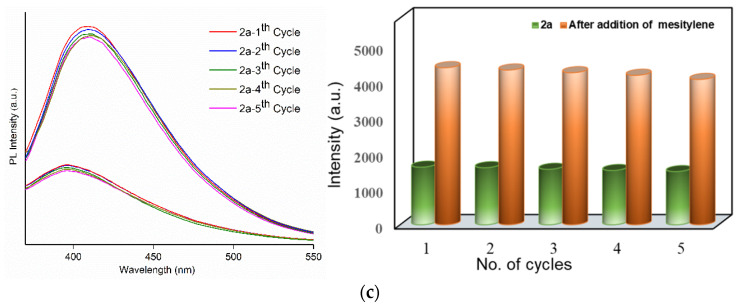
(**a**) Emission spectra of activated **1** (left) and **2** (right) loaded with different VOC’s. Emission spectra showing the recyclability of (**b**) **1a** and (**c**) **2a** exposed with mesitylene for five repeated cycles.

The CO_2_ gas adsorption studies confirm the permanent pores in the eight-fold interpenetrating CPs **1** and **2**, which provide a unique opportunity to investigate the role of the entangled bpba-CPs in the sensing of abundant VOCs [26,27,28,29,30,31,32] and metal ions [33]. The activated complexes of **1** and **2**, **1a** and **2a**, were exposed to various VOCs by immersing grounded samples (30 mg) into 5 mL different organic solvents, which were then sonicated for 10 min and collected by centrifugation and then air dried. The emission spectra of the VOC-absorbed complexes upon excitation at 350 nm were measured at room temperature (Figure 4a), revealing that the emission intensities of VOC-absorbed **1a** and **2a** reached the maximum for the mesitylene molecules. The PXRD patterns shown in Figure S16 for the mesitylene absorbed **1a** and **2a** indicate that they maintain the identical structural frameworks. The luminescence enhancement, [(I − I_o_)/I_o_] (I_o_ = luminescence intensity of **1a** or **2a**; I = luminescence intensity of mesitylene-loaded **1a** or **2a**) due to the adsorption of mesitylene are calculated as 1.91 and 1.65 for **1a** and **2a**, respectively. The organic uptakes were estimated from the weight-losses in TGA curves of VOC-loaded **1a** and **2a** (Figure S17), showing that the uptakes of mesitylene molecules per unit cell are 2.53 in **1a** and 2.97 in **2a**, respectively. The emission wavelengths of mesitylene-absorbed **1a** and **2a** shows red-shifts of 13 and 11 nm, respectively, compared with solvent-free **1a** and **2a** (Figures S18a and S19a), indicating the possible interligand couplings [34]. The mesitylene molecules may have the strongest C-H···π interactions with **L** linkers among other exposed solvents, inducing the largest bathochromic shift and the intensity enhancement [35]. Recently, it has been shown that C-H···π interaction can be modulated to enhance the luminescence [36]. 

To estimate the selective sensing performances of **1a** and **2a** toward mesitylene, luminescence titration experiments were performed by the addition of the increasing mesitylene (0–5 mL) to **1a** and **2a**. The volume of mesitylene and the emission intensity show linear dependence of R^2^ = 0.983 and 0.985 (Figures S18b,c and S19b,c). Table S1 shows the intensity enhancements of several reported complexes due to the interactions with the specific organic solvents, showing comparable luminescence enhancements for **1a** and **2a** in the detection of mesitylene molecules. Further, to determine the recyclability of mesitylene adsorption in complexes **1a** and **2a**, their luminescence sensing abilities were explored for five regeneration cycles. The PXRD patterns in Figure S16 indicate that the main diffraction peaks of **1a** and **2a** are quite identical with those of the mesitylene-treated samples, while Figure 4b,c display their similar emission spectra and intensities, respectively, suggesting that their frameworks remain rigid for five cycles. 

### 3.8. Detection of Metal Ions 

**Figure 5:**
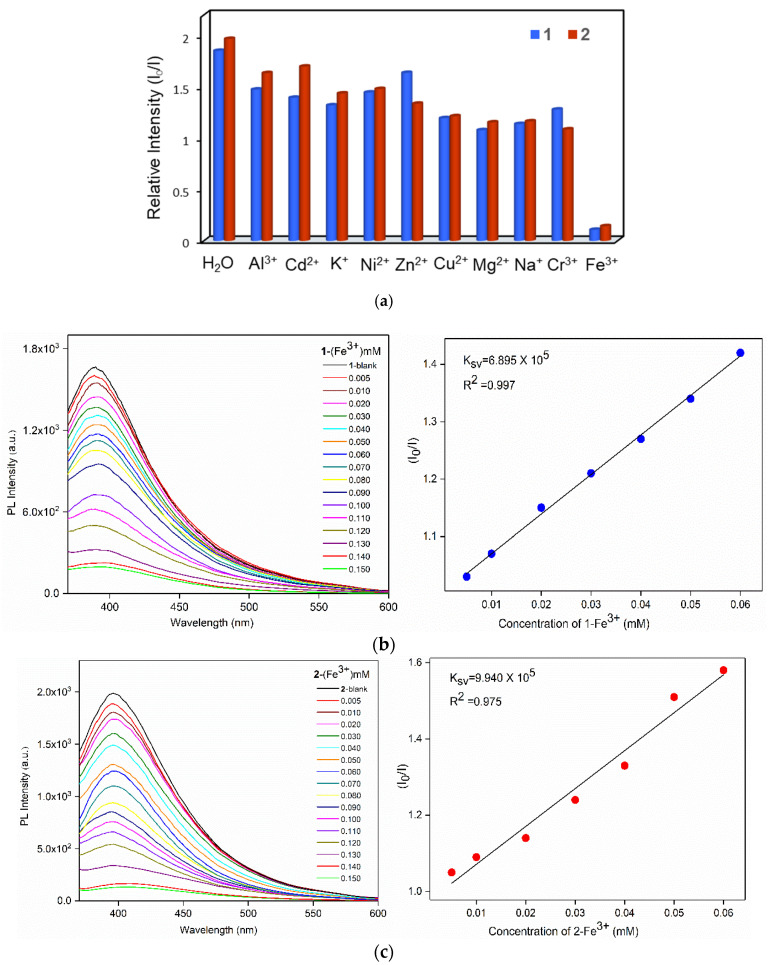
(**a**) Bar diagrams showing the relative emission intensities of **1** and **2** in various metal ions. Emission spectra of the activated (**b**) **1** and (**c**) **2** at various concentrations of Fe^3+^ (left), and the Stern−Volmer (sv) plot of I_0_/I versus Fe^3+^ concentration (right).

In order to explore the potential application of **1** and **2** in luminescent sensing of metal cations, 30 mg samples of **1** and **2**, respectively, were immersed into 10 mL aqueous solutions of nitrate salts M(NO_3_)_x_ (M = Al^3+^, Cd^2+^, Cu^2+^, Mg^2+^, Ni^2+^, Zn^2+^, Na^+^, K^+^, Cr^3+^ and Fe^3+^) with a concentration of 1 × 10^−3^ M. After 1 h, the solids were filtered and then the solid-state emission spectra were measured at room temperature. As shown in Figure S20, remarkable luminescence quenching of about 95% for **1** and 92% for **2** were found in the detection of Fe^3+^ ions. To further explore the quenching effect of Fe^3+^ ions, sensing dependence of luminescence intensity on the concentration of Fe^3+^ was investigated by immersing finely grounded samples (30 mg) of **1** and **2** into Fe^3+^ aqueous solutions with various concentrations (0.005–0.15 mM) for 1 h. As shown in Figure 5b,c (left), the emission intensities were getting lower and almost completely quenched upon increasing the concentration of Fe^3+^. Quantitatively, the quenching capacity of Fe^3+^ ion can be rationalized by the Stern–Volmer equation: I_0_/I = 1 + K_sv_ × [Q], where [Q] is the concentration of Fe^3+^, K_sv_ is the quenching constant, and I_0_ and I are the emission intensities in the absence and presence of Fe^3+^, respectively [37]. As demonstrated in Figure 5b,c (right), the titration curves for Fe^3+^ ions in **1** and **2** are virtually linear at low concentrations, which gave the linear correlation coefficient (R^2^) of 0.997 for **1** and 0.975 for **2**, respectively, while the S-V curves at higher concentrations became nonlinear, which can be described by concurrent dynamic and static quenching or the self-absorption process. The Stern−Volmer constant (K_sv_) values are 6.895 × 10^5^ for **1** and 9.940 × 10^5^ M^−1^ for **2**, respectively, in the presence of Fe^3+^. 

Furthermore, the detection limits were calculated according to the standard equation 3*σ*/*k*, where σ is the standard deviation from the blank measurements and *k* is the absolute value of the calibration curve at lower concentration [38], giving 2.35 and 1.01 μM for **1** and **2**, respectively. Noticeably, complexes **1** and **2** show comparative K_sv_ with some previously reported complexes toward the detection of Fe^3+^ ions in aqueous phase solution (Table S2). The recyclability test showed no significant differences in the PXRD patterns (Figure S21) and the luminescence intensities (Figure S22) for five regeneration cycles, indicating the reusability and demonstrating that the quenching effect was not due to the structural decomposition of **1** and **2** upon the interactions with the Fe^3+^ ions. 

Several mechanisms for luminescence quenching such as framework collapse, cation exchange, and interactions between the incoming metal ion and the organic linker that result in competitive absorption of the excited energies of the sensed metal ion and the host CPs, have been suggested [39]. As mentioned above, by comparing the PXRD patterns and luminescence intensities, the framework collapse can be ruled out. The interactions between Fe^3+^ ions and complexes **1** and **2** through the amide carbonyl oxygen atoms may thus lead to the luminescence quenching [40]. The UV-vis absorption spectrum of Fe^3+^ in aqueous solution and the corresponding excitation and emission spectra of complexes **1** and **2** are shown in Figure S23. Partial overlaps between the absorption spectrum of Fe^3+^ ion and the excitation spectra of complexes **1** and **2** are observed, indicating that the excitation energies of **1** and **2** can be partially absorbed by the Fe^3+^ ions, and the luminescence quenching can most probably be ascribed to competitive energy absorption [39]. 

## 4. Conclusions

Three eight-fold interpenetrated 3D CPs with the (6^6^)-**dia** topology have been successfully prepared by using angular **L** and the linear dicarboxylic acid 1,4-H_2_NDC. The Zn(II), Cd(II) and Co(II) metal centers of **1**–**3** adopt the distorted tetrahedral, pentagonal bipyramidal and octahedral/square pyramidal geometries, respectively, resulting in different coordination modes for the 1,4-NDC^2−^ ligands and indicating that the structural topology is not subject to the metal geometry. Their structural stabilities are assessed particularly in the aqueous environments, revealing that complexes **1** and **2** with close-shell Zn(II) and Cd(II) ions are much more stable than **3** with the open-shell Co(II) ion. The surface areas and pore structures of **1** and **2** were observed via CO_2_ gas sorption measurements and the activated samples of **1** and **2** explored by emission spectra and PXRD patterns demonstrate efficient detection of mesitylene and Fe^3+^ ions with high selectivity and recyclability. This investigation oversees the sensing properties of the highly entangled bpba-based CPs toward VOCs and metal ions. 

## Figures and Tables

**Table 1:** Crystal data for complexes **1**–**3**.

Complex	1	2	3
Formula Formula weight	C_36_H_26_ZnN_4_O_8_ 707.98	C_37_H_30_CdN_4_O_9_ 787.05	C_37_H_29_CoN_4_O_8.5_ 724.57
Crystal system	monoclinic	monoclinic	monoclinic
Space group	*P*2_1_/*c*	*P*2_1_/*n*	*P*2_1_/*n*
a, Å	9.78070(1)	8.5591(4)	8.5240(6)
b, Å	19.3224(2)	21.5741(11)	21.5176(16)
c, Å	17.9006(2)	19.0086(9)	18.1376(13)
α, °	90	90	90
β, °	103.1124(8)	98.065(3)	96.850(2)
γ, °	90	90	90
V, Å^3^	3294.77(6)	3475.3(3)	3303.0(4)
Z	4	4	4
D_calc_, mg/m^3^	1.427	1.504	1.457
F(000)	1456	1600	1496
µ (MoK_α_), mm^−1^	0.805	0.690	0.582
Range (2θ) for data collection, deg.	3.14 ≤ 2θ ≤ 52.00	2.87 ≤ 2θ ≤ 52.00	4.41 ≤ 2θ ≤ 50.12
Independent reflections	6474 [R(int) = 0.0398]	6845 [R(int) = 0.0633]	5855 [R(int) = 0.1880]
Data/restraints/parameters	6474/0/437	6845/1/496	5855/0/487
Quality-of-fit indicator ^a^	1.034	1.076	1.027
Final R indices [I > 2σ(I)] ^b,c^	R1 = 0.0618, wR2 = 0.1761	R1 = 0.0648, wR2 = 0.1609	R1 = 0.0655, wR2 = 0.1477
R indices (all data)	R1 = 0.0871 wR2 = 0.1939	R1 = 0.0935 wR2 = 0.1751	R1 = 0.1986 wR2 = 0.2248

^a^ quality-of-fit = [Σw(|F_o_^2^| − |F_c_^2^|)^2^/N_observed_ − N_parameters_)]^1/2^. ^b^ R_1_ = Σ||F_o_| − |F_c_||/Σ|F_o_|. ^c^ wR_2_ = [Σw(F_o_^2^ − F_c_^2^)^2^/Σ(F_o_^2^)^2^]^1/2^. w = 1/[σ^2^(F_o_^2^) + (ap)^2^ + (bp)], p = [max(F_o_^2^ or 0) + 2(F_c_^2^)]/3. **1**, a = 0.1067, b = 3.1664; **2**, a = 0.0754, b = 7.0332; **3**, a = 0.0849, b = 3.8585.

**Table 2:** Selected angles of the **L** ligands in complexes **1**–**3**.

Complex	C-O-C Angle (°)	Dihedral Angle (°)		
		d1	d2	d3
**1**	117.0	30.49	74.52	21.12
**2**	116.9	25.51	87.63	35.97
**3**	116.9	28.54	88.70	34.02

**Table 3:** Thermal properties of **1**–**3**.

Complex.	Weight Loss of Solvents °C (found/calc), %	Weight Loss of Ligands °C (found/calc), %
**1**	~120 (2.76/2.54)	240–800 (84.89/88.21)
**2**	~140 (3.33/4.07)	250–800 (80.83/81.90)
**3**	~145 (3.25/4.42)	300–800 (87.34/87.64)

**Table 4:** Luminescent properties of **L**, 1,4-H_2_NDC, **1** and **1a**, **2** and **2a**.

Compound	Excitation λ_ex_ (nm)	Emission λ_em_ (nm)	Compound	Excitation λ_ex_ (nm)	Emission λ_em_ (nm)
**L**	376	468	1,4-H_2_NDC	280, 370	480
**1**	350	390	**1a**	350	390
**2**	350	395	**2a**	350	395

## Supplementary Materials

The following are available online at https://www.mdpi.com/article/10.3390/polym13183018/s1. FT-IR spectra for **1**–**3** (Figure S1). PXRD patterns for **1**–**3** (Figures S2–S4). TGA curves for **1**–**3** (Figure S5). CO_2_ sorption isotherms of **1** and **2** (Figures S11–S13). Emission spectra for **1** and **2** (Figures S14 and S15). Sensing properties of reported compounds toward various aromatic VOCs (Table S1). Sensing properties of reported compounds toward Fe^3+^ ions (Table S2). Crystallographic data for **1**–**3** have been deposited with the Cambridge Crystallographic Data Centre, CCDC No. 2100027-2100029.
