# Supplementary material for: Eight-Fold Interpenetrating Diamondoid Coordination Polymers for Sensing Volatile Organic Compounds and Metal Ions
Source: Polymers (Basel). 2021 Sep 7;13(18):3018. doi: 10.3390/polym13183018 (PMC8470056; doi:10.3390/polym13183018)
Supplement: Supplementary file 1 [file polymers-13-03018-s001.zip › polymers-1345810-supplementary.pdf]

Supplementary Information

Eight-Fold Interpenetrating Diamondoid Coordination Polymers for Sensing Volatile Organic Compounds and Metal Ions

Venkatesan Lakshmanan [1], Yi-Ting Lai [1], Xiang-Kai Yang [1], Manivannan Govindaraj [1], Chia-Her Lin [2,*] and Jhy-Der Chen [1,*]

[1] Department of Chemistry, Chung-Yuan Christian University, Chung Li 32023, Taiwan; venkatesanflower95@gmail.com (V.L.); e0912583362@gmail.com (Y.-T.L.); xiangkaishulin@gmail.com (X.-K.Y.); manivannanjent@gmail.com (M.G.)
[2] Department of Chemistry, National Taiwan Normal University, Taipei 11677, Taiwan
* Correspondence: chiaher@ntnu.edu.tw (C.-H.L.); jdchen@cycu.edu.tw (J.-D.C.); Tel.: +886-3-265-3351 (J.-D.C.)

Citation: Lakshmanan, V.; Lai, Y.-T.; Yang, X.-K.; Govindaraj, M.; Lin, C.-H. Eight-Fold Interpenetrating Diamondoid Coordination Polymers for Sensing Volatile Organic Compounds and Metal Ions. *Polymers* **2021**, *13*, x. https://doi.org/10.3390/xxxxx

Academic Editor:

Received: date
Accepted: date
Published: date

Publisher's Note: MDPI stays neutral with regard to jurisdictional claims in published maps and institutional affiliations.

Copyright: © 2021 by the authors. Submitted for possible open access publication under the terms and conditions of the Creative Commons Attribution (CC BY) license (http://creativecommons.org/licenses/by/4.0/).

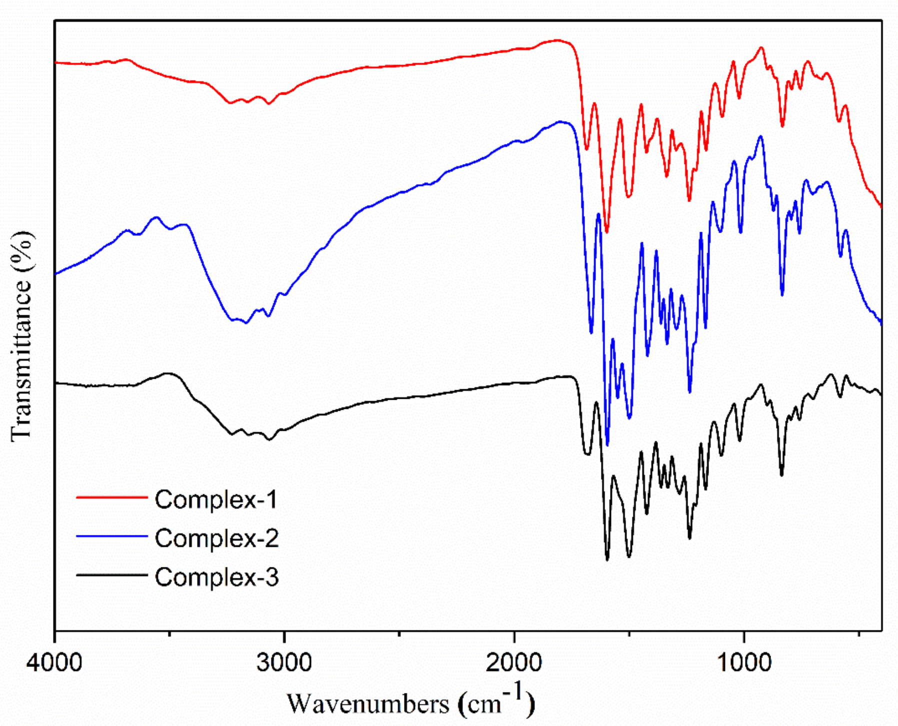

Figure S1. FT-IR spectra of complexes 1 - 3.

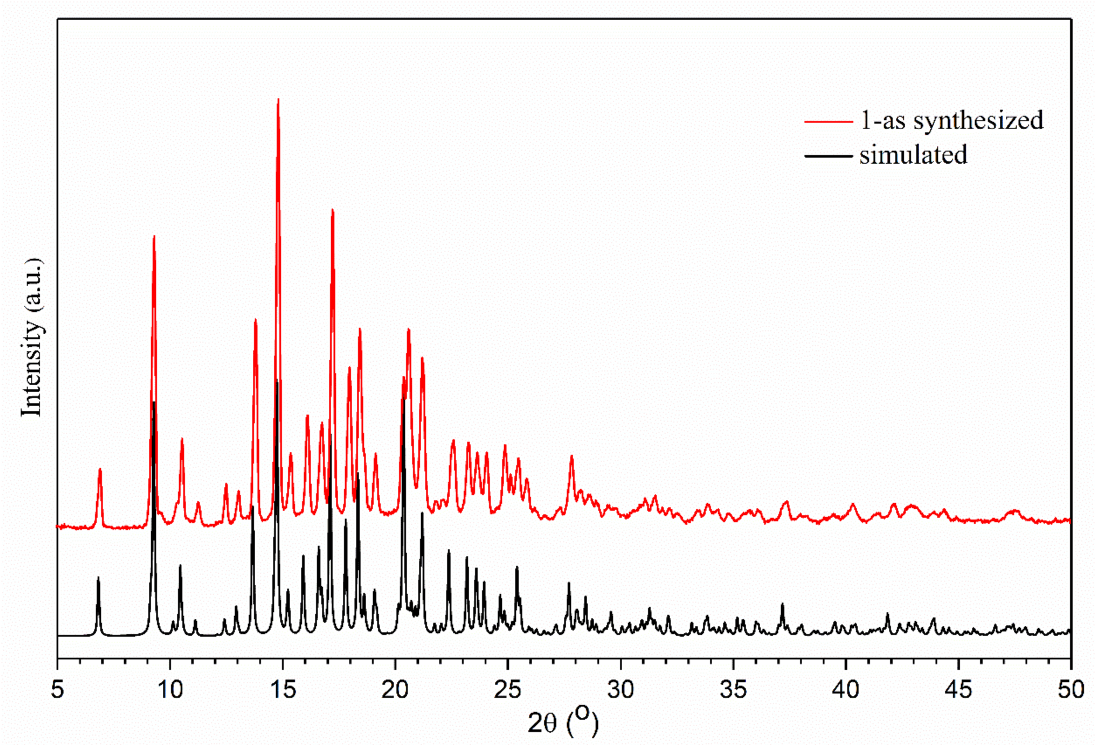

Figure S2. PXRD patterns of 1.

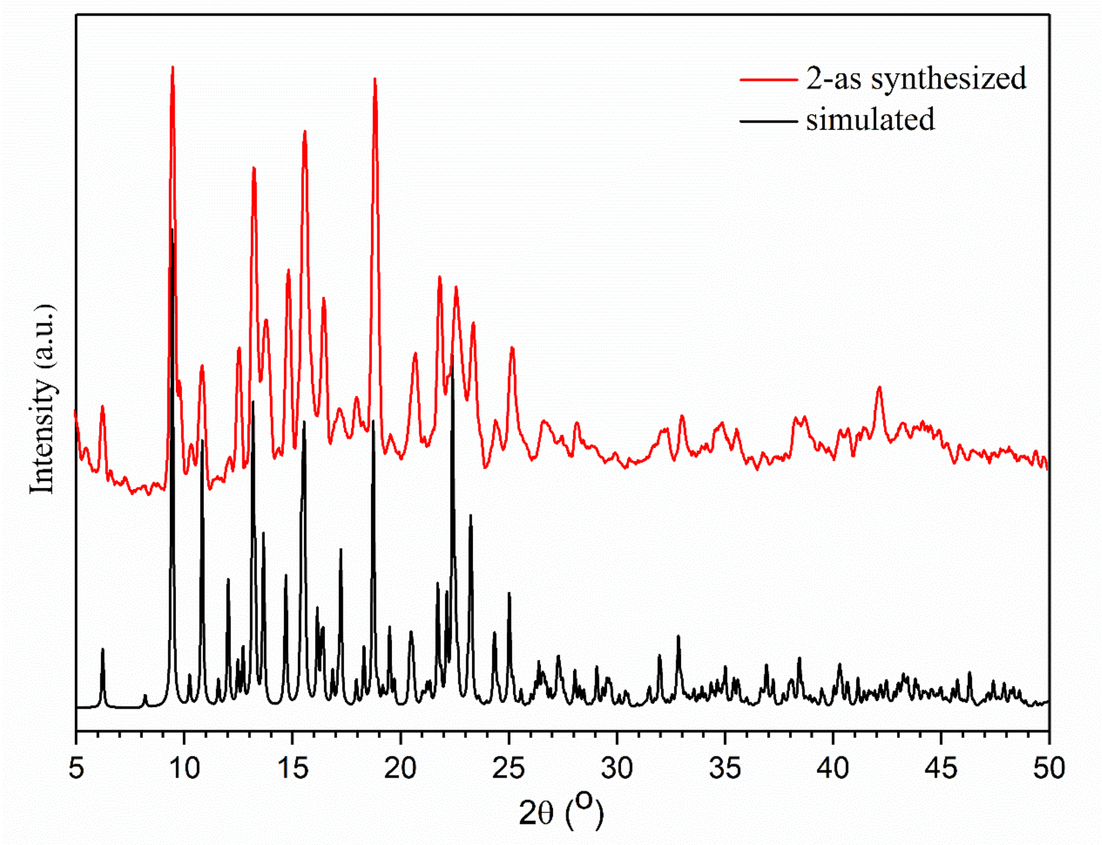

Figure S3. PXRD patterns of **2**.

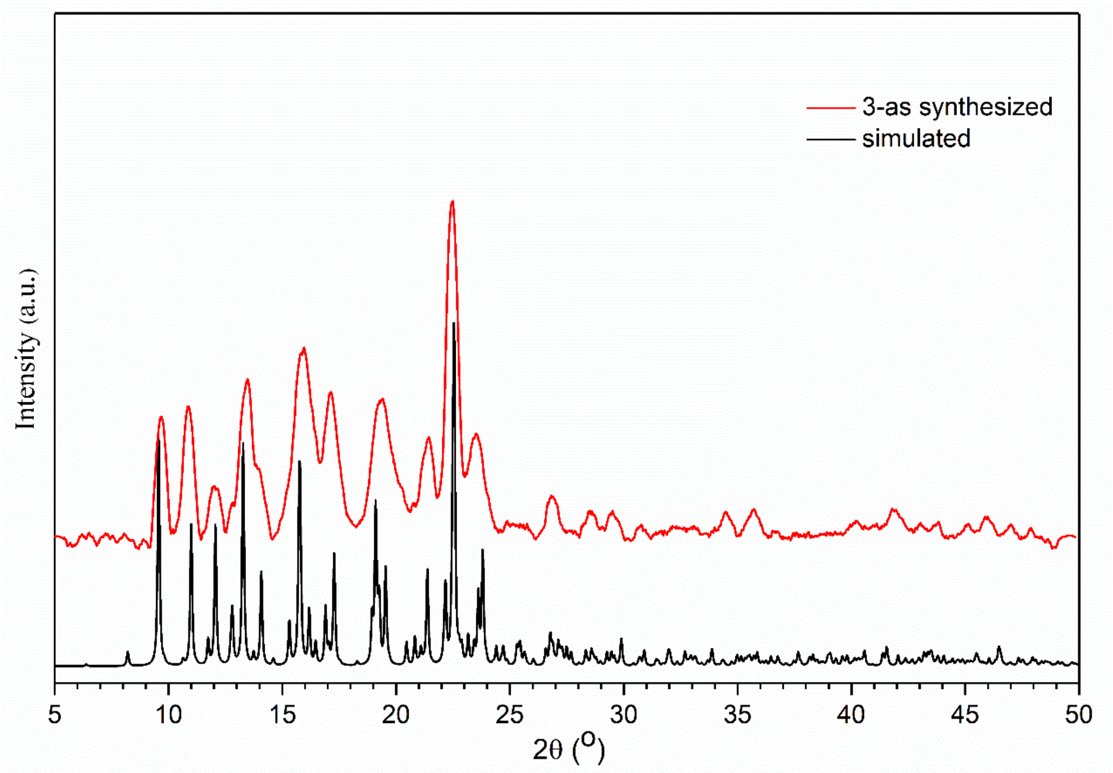

Figure S4. PXRD patterns of **3**.

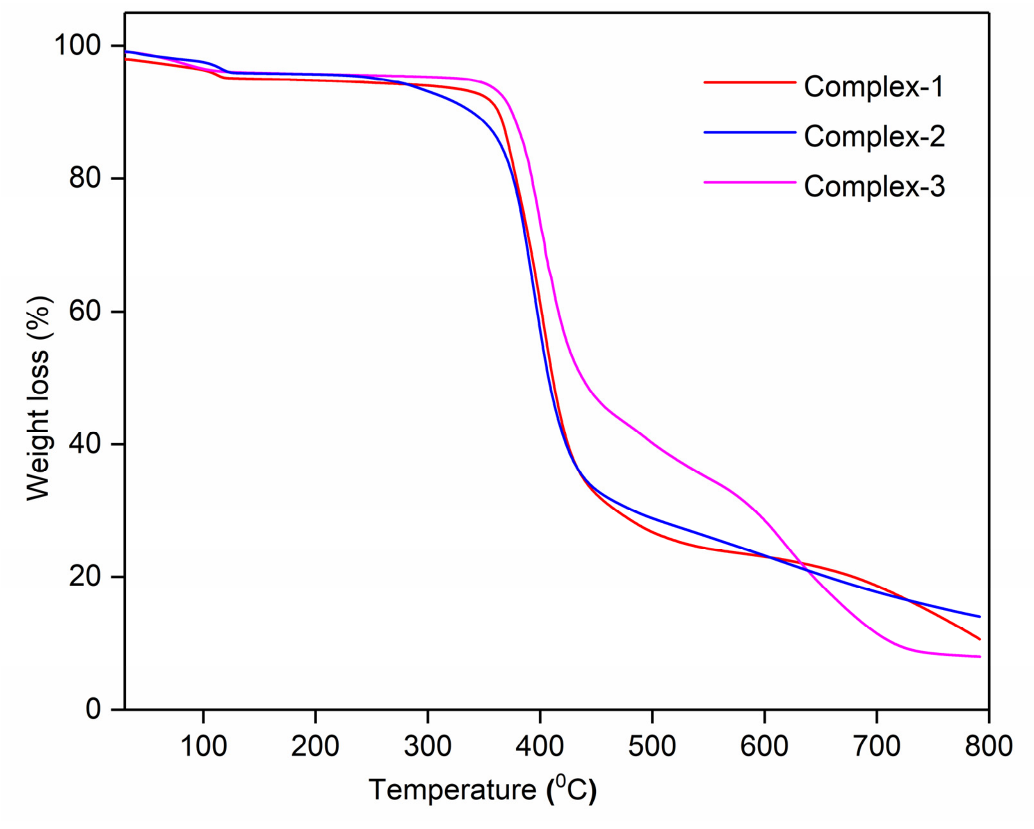

Figure S5. TGA curves for complexes **1** (red), **2** (blue) and **3** (magenta).

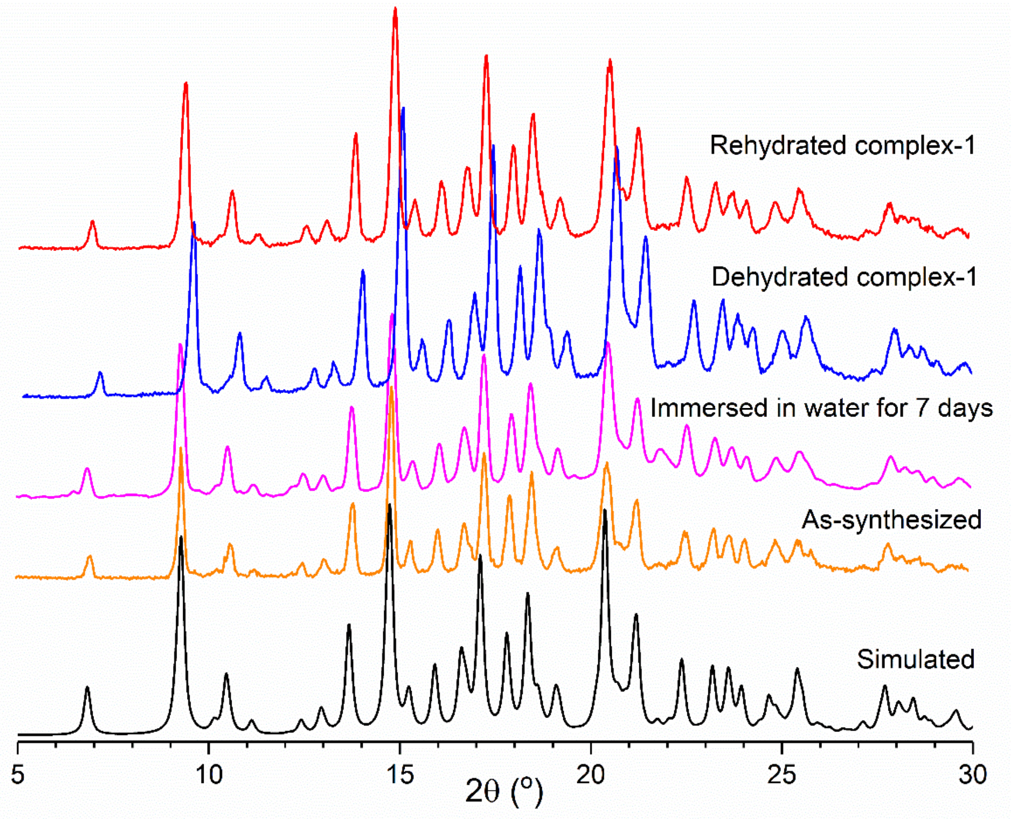

Figure S6. PXRD patterns of the dehydrated and rehydrated **1**.

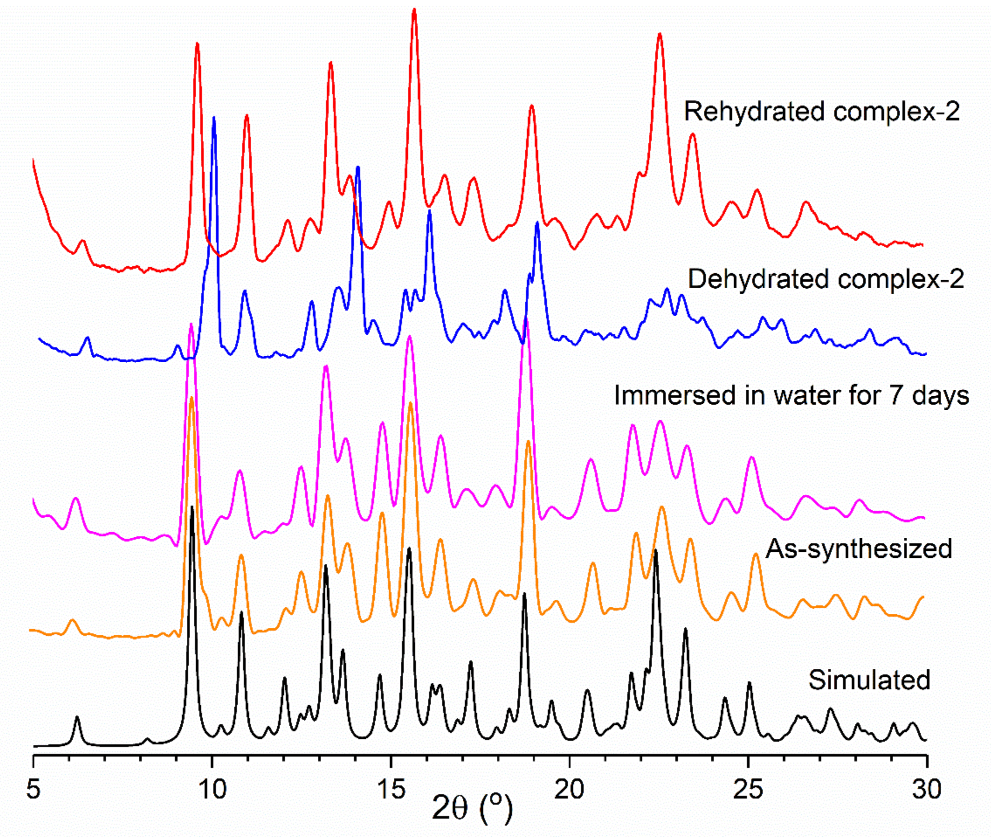

Figure S7. PXRD patterns of the dehydrated and rehydrated **2**.

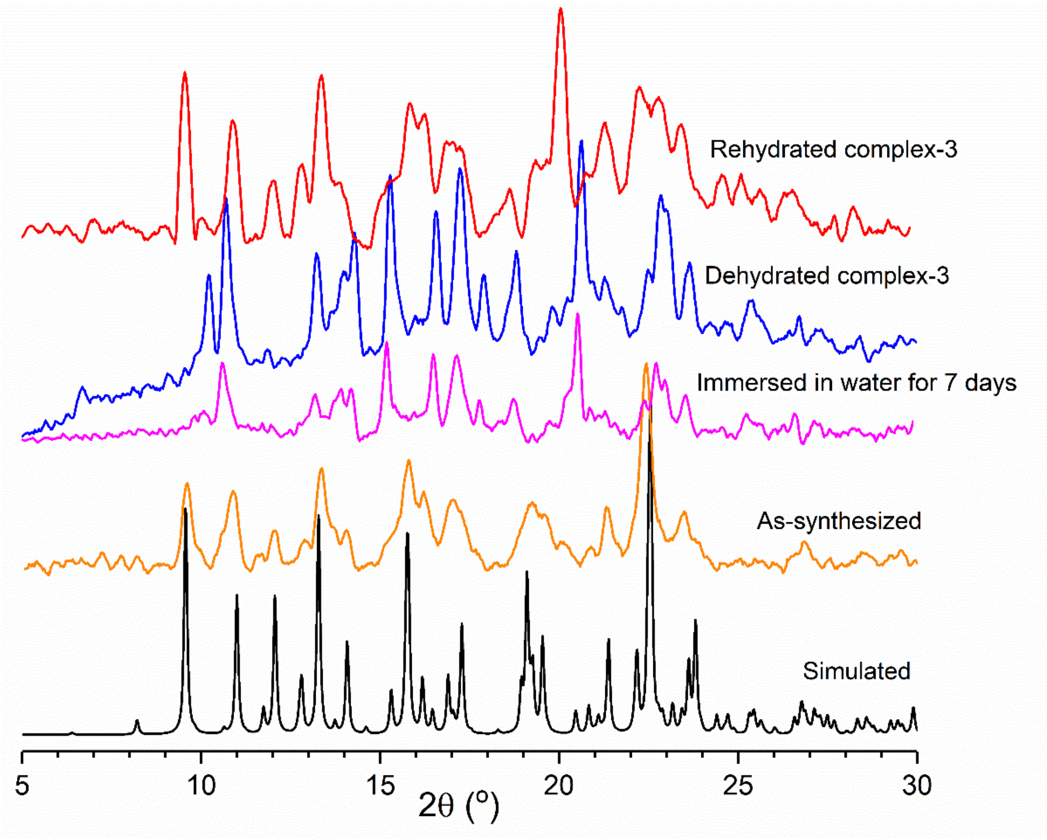

Figure S8. PXRD patterns of the dehydrated and rehydrated **3**.

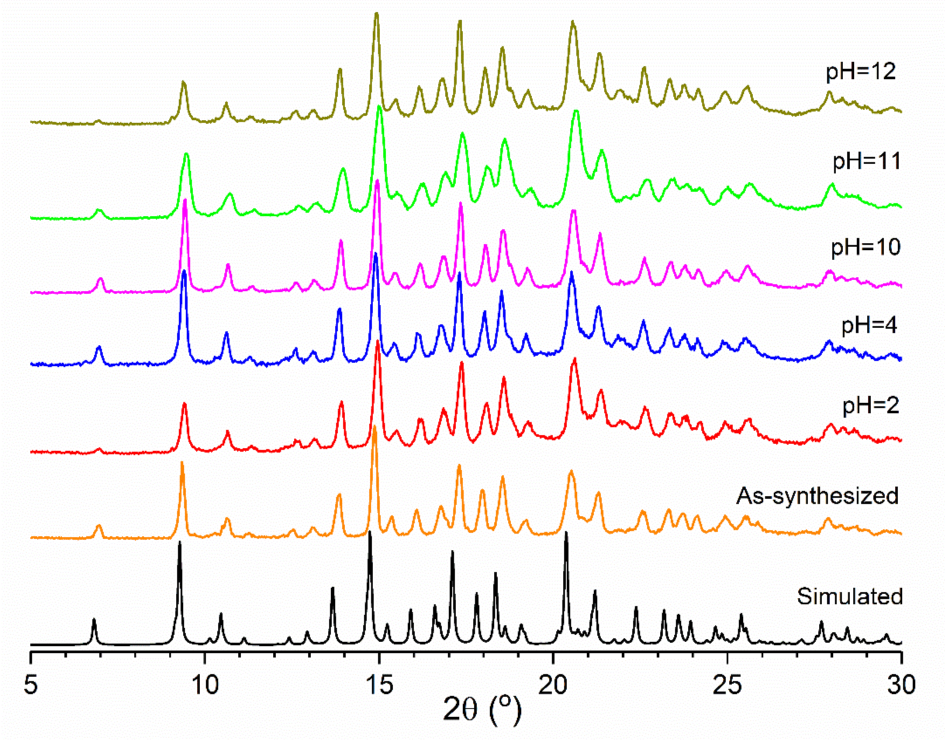

Figure S9. PXRD patterns of complex **1** in various pH values.

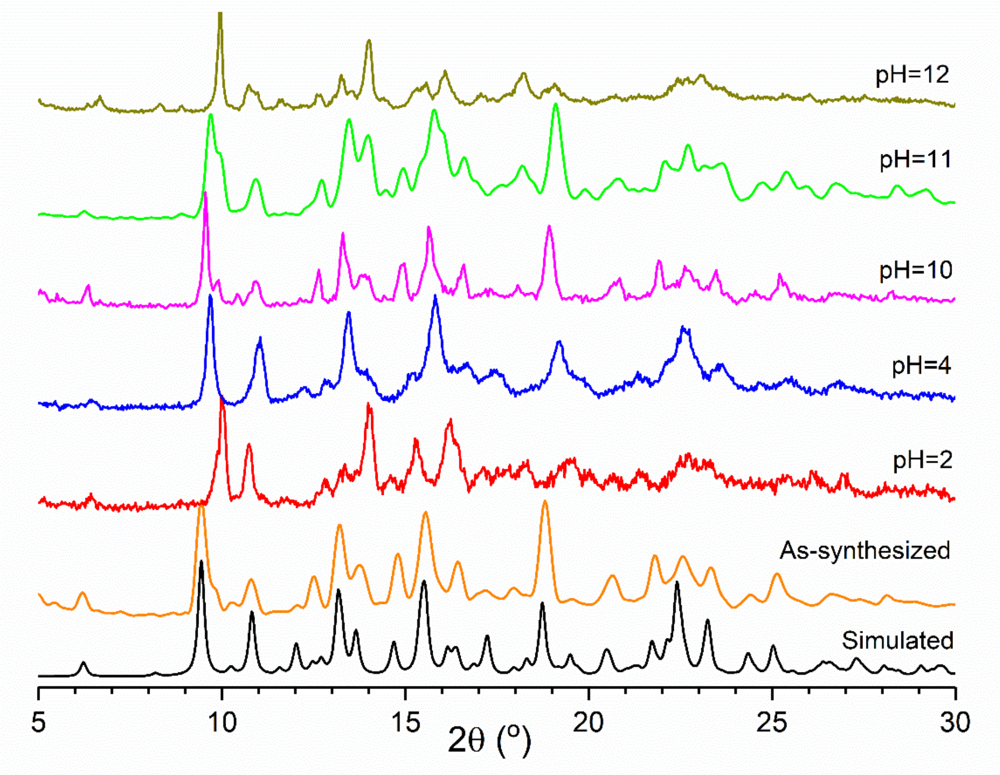

Figure S10. PXRD patterns of complex **2** in various pH values.

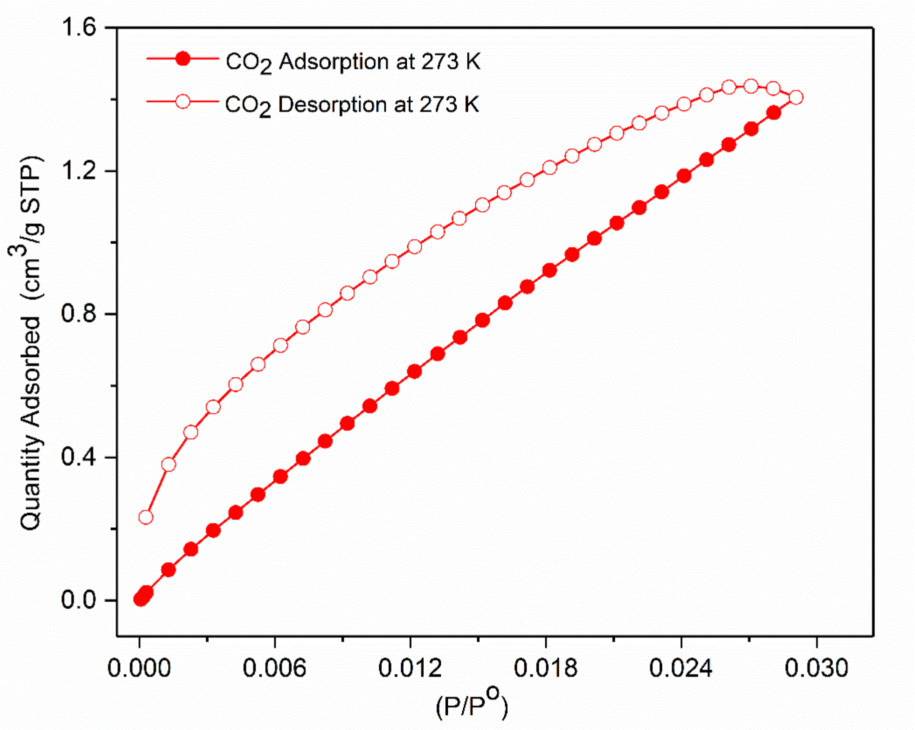

(a).

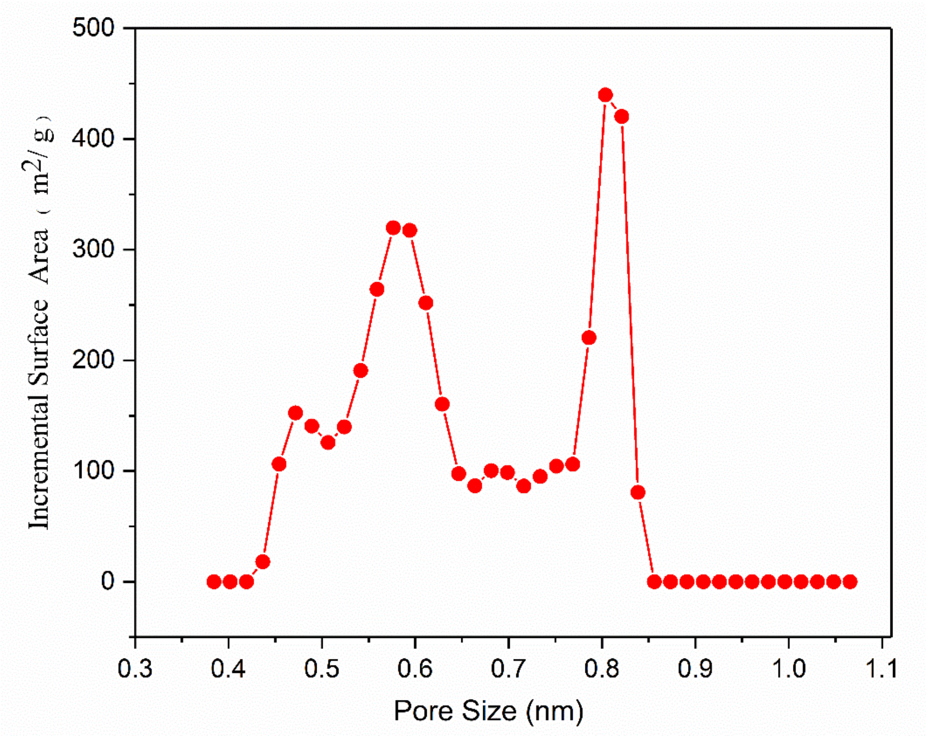

(b).

Figure 11. (**a**) CO_2 sorption isotherm of complex **1** at 273 K and (**b**) pore size distributions.

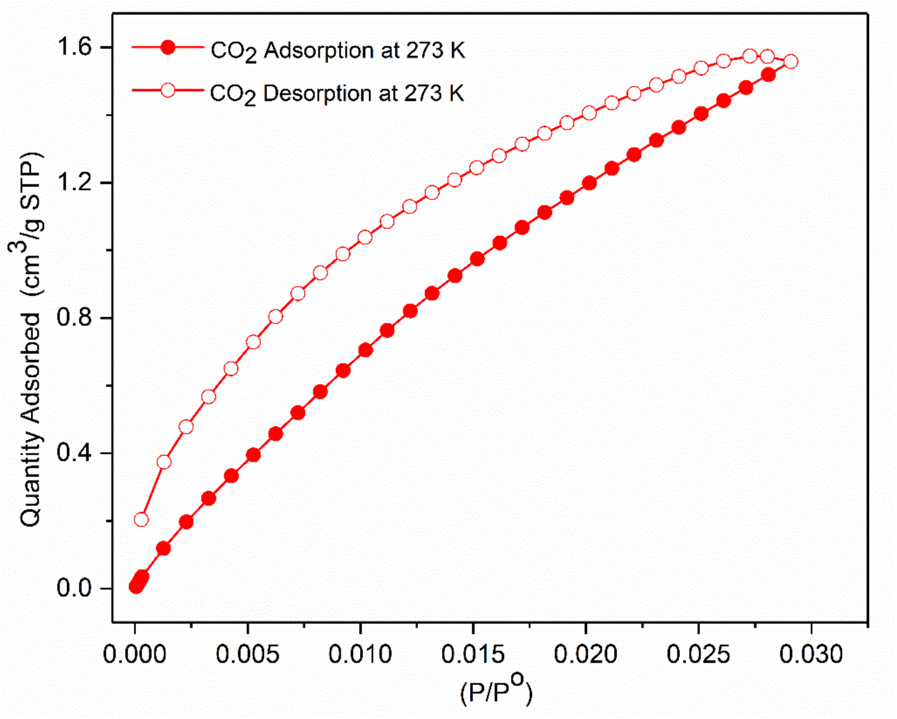

(a).

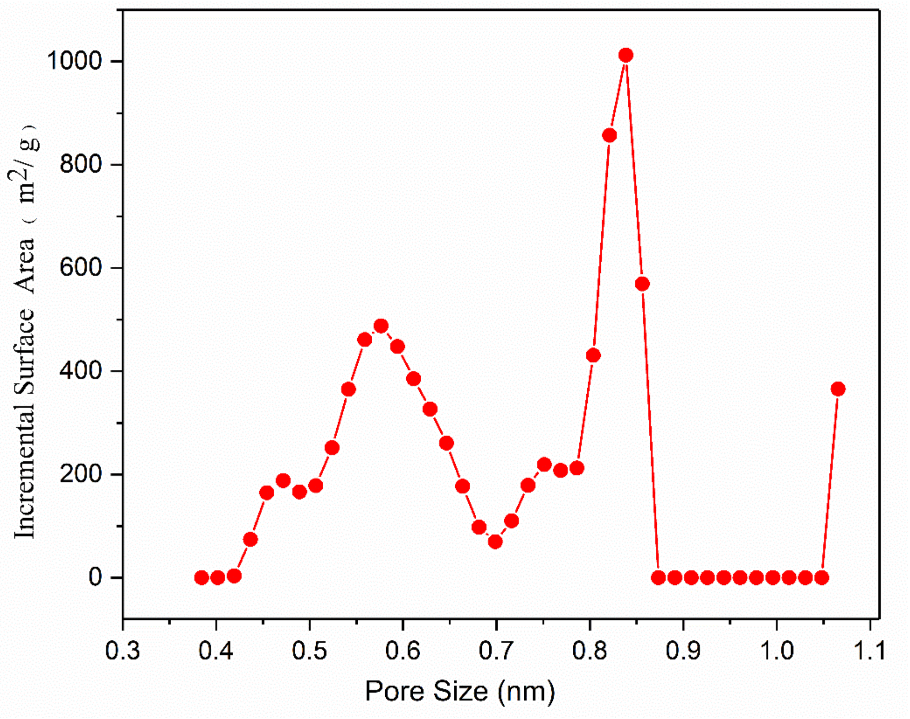

(b).

Figure S12. (**a**) CO_2 sorption isotherm of complex **2** at 273 K and (**b**) pore size distributions.

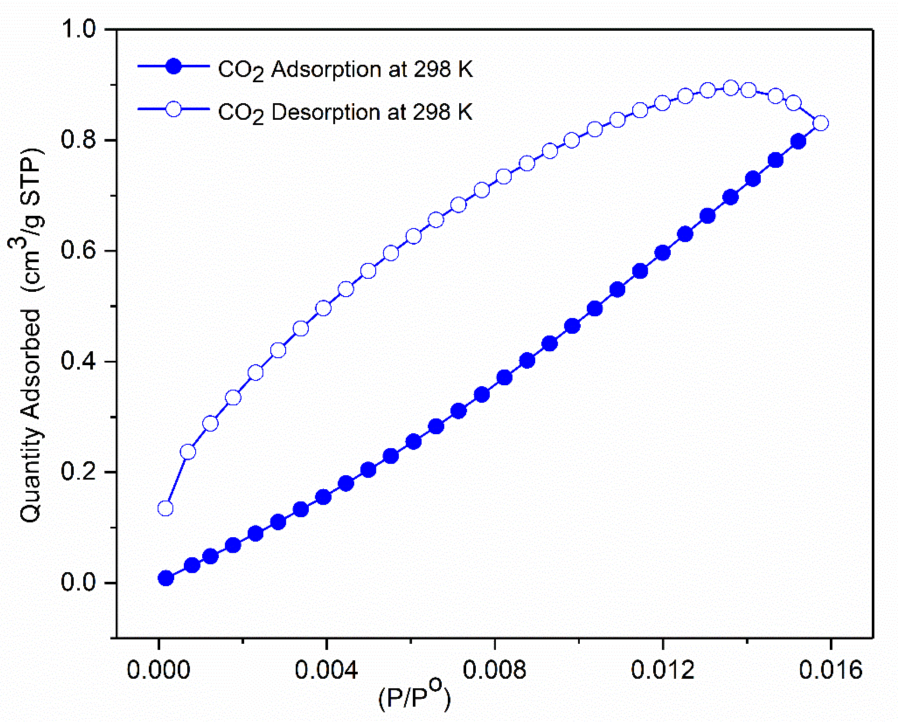

(a).

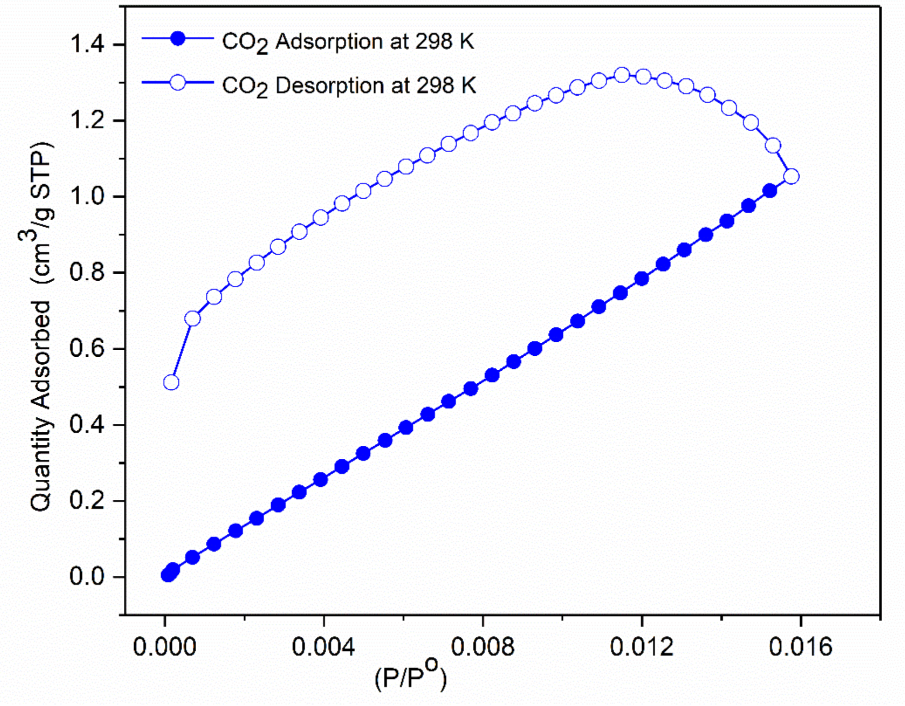
CO2 Adsorption at 298 K
CO2 Desorption at 298 K
Quantity Adsorbed (cm3/g STP)
(P/Po)
1.4
1.2
1.0
0.8
0.6
0.4
0.2
0.0
0.000
0.004
0.008
0.012
0.016

(b).

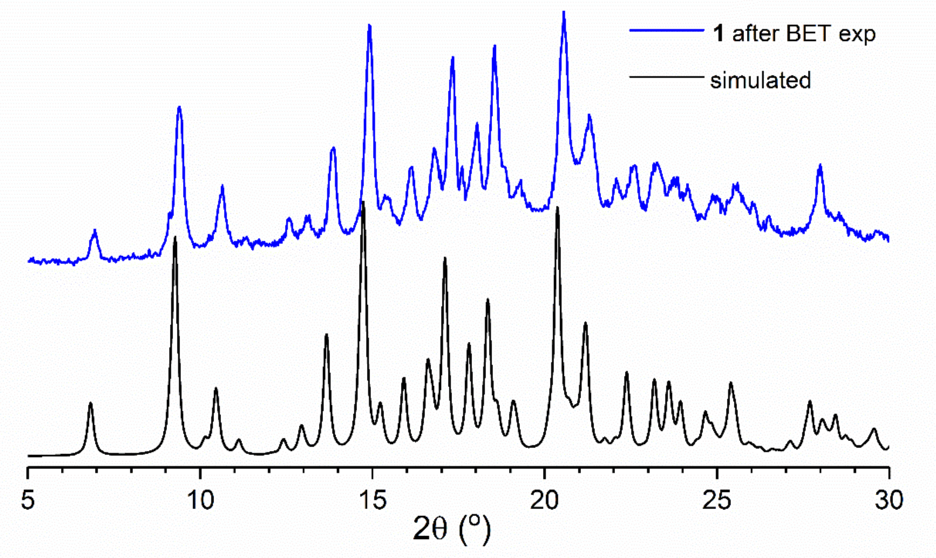
1 after BET exp
simulated
2θ (°)
5
10
15
20
25
30

(c).

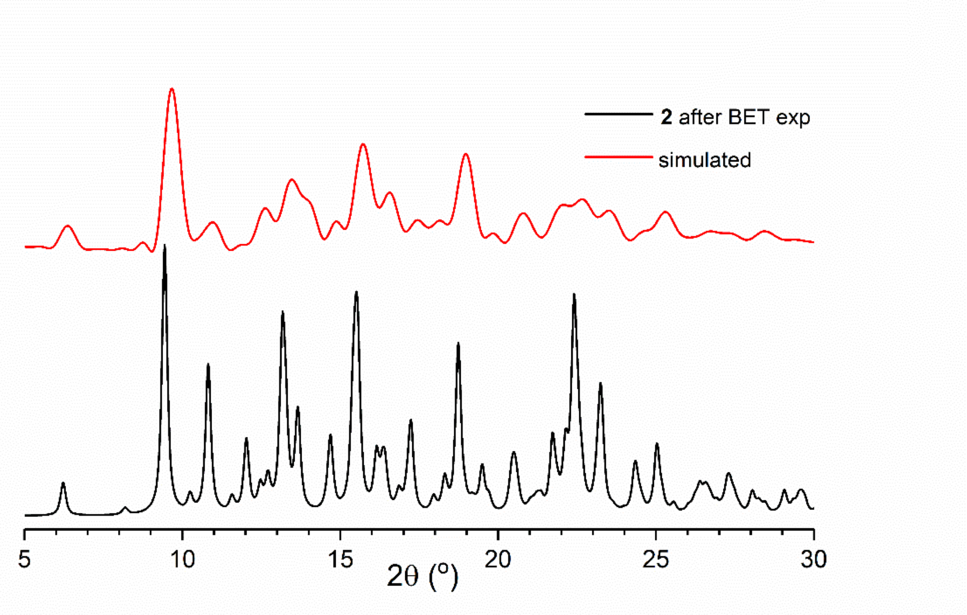

(d).

Figure S13. CO_2 sorption isotherms of complexes (**a**) **1** and (**b**) **2** at 298 K and PXRD patterns of (**c**) **1** and (**d**) **2** measured after experiments.

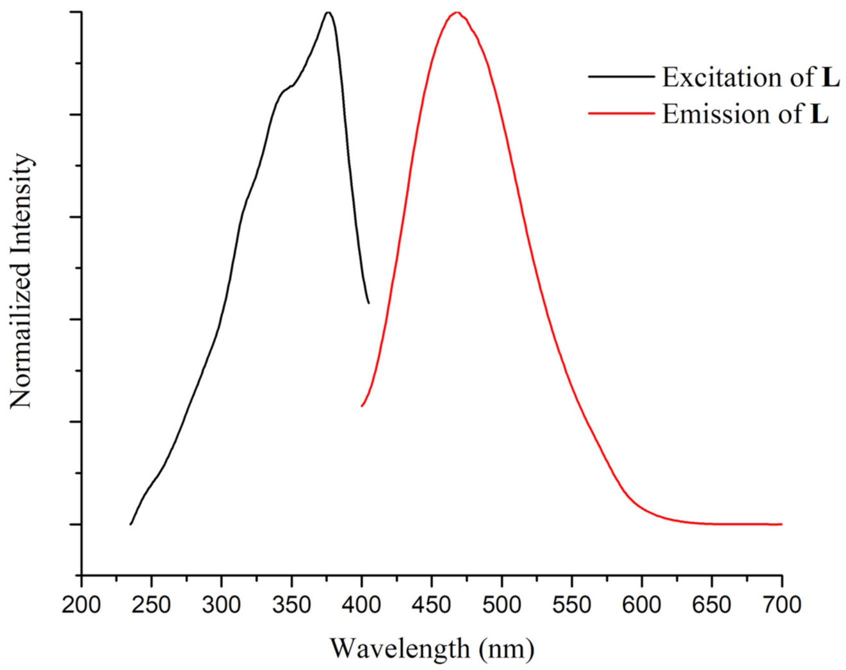

(a).

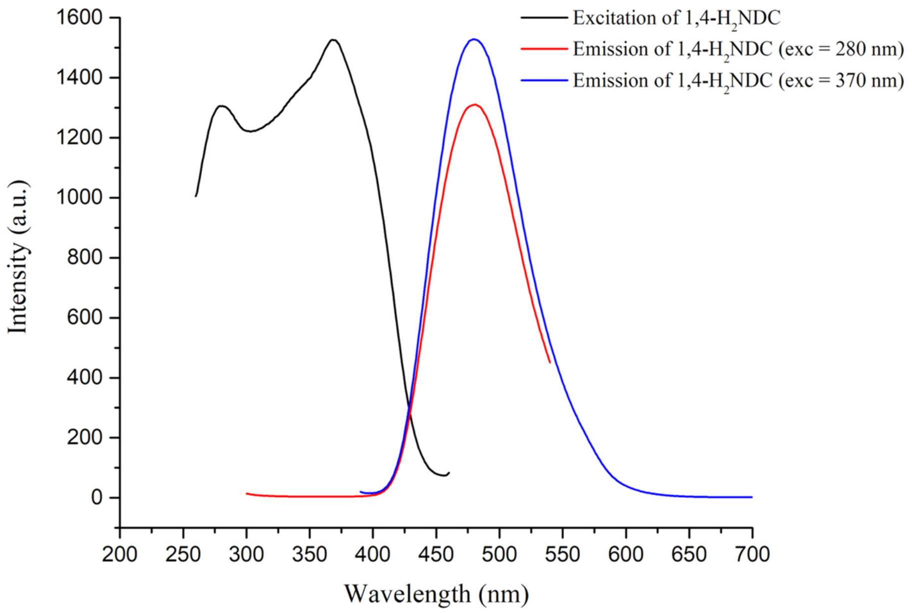

(b).

Figure S14. (**a**) Emission spectra of ligand **L** and (**b**) emission spectra of 1,4-H2NDC.

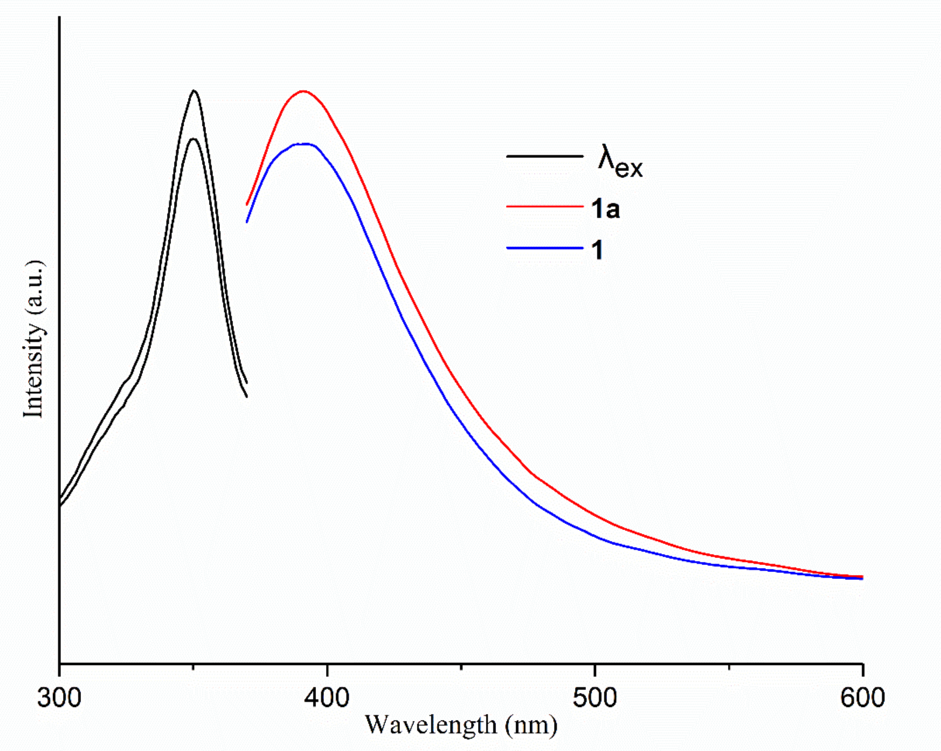

(a).

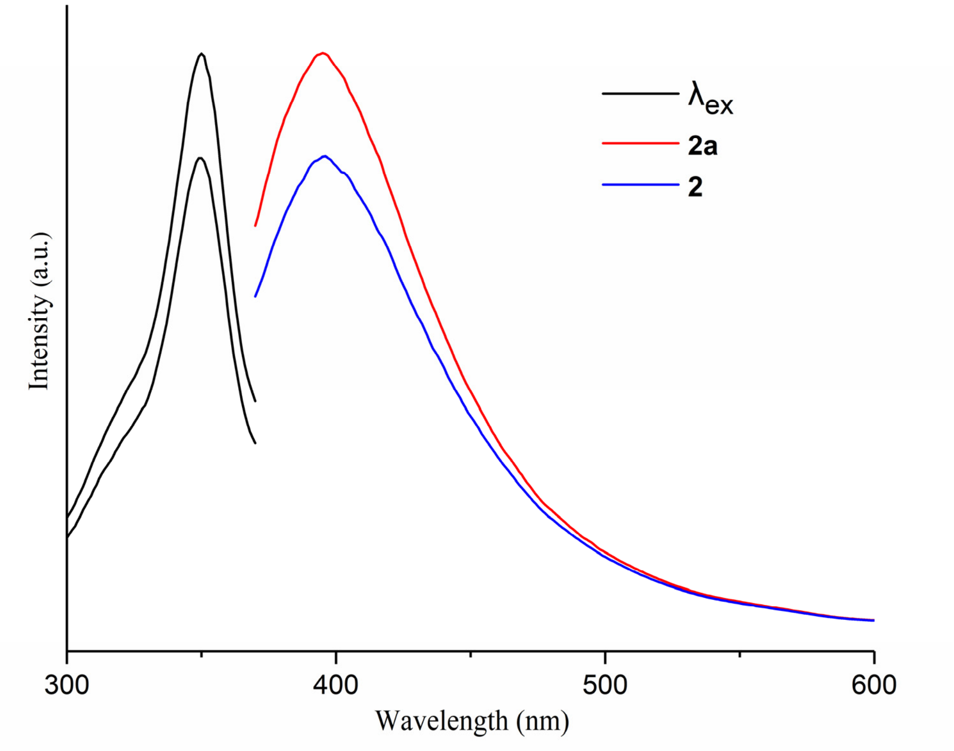

(b).

Figure S15. Emission spectra of (**a**) complex **1** and **1a** and (**b**) complex **2** and **2a**.

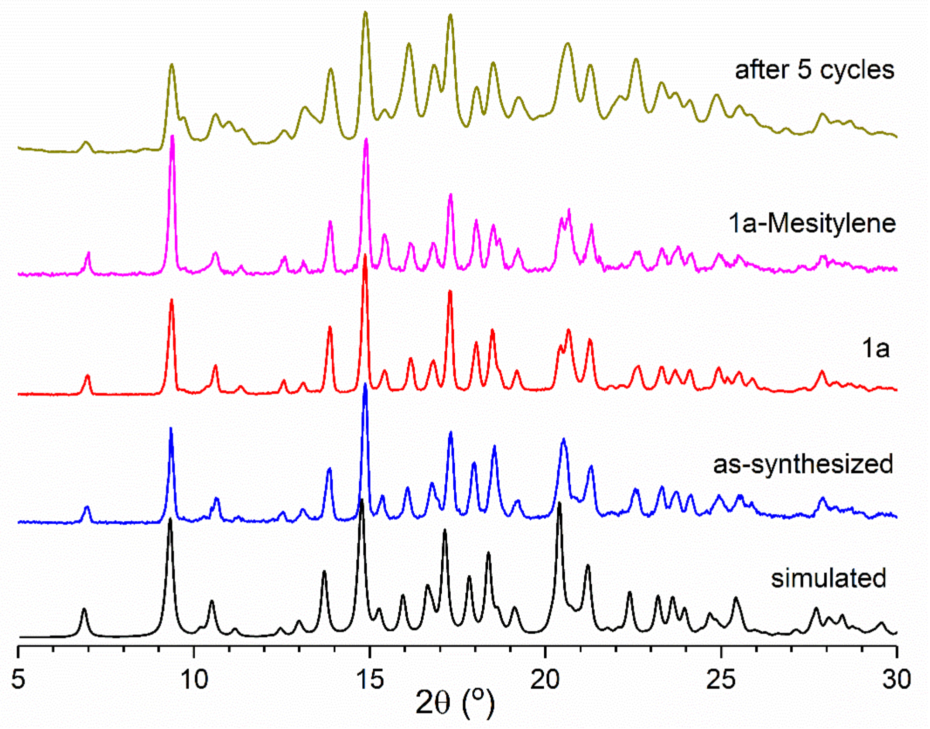

(a).

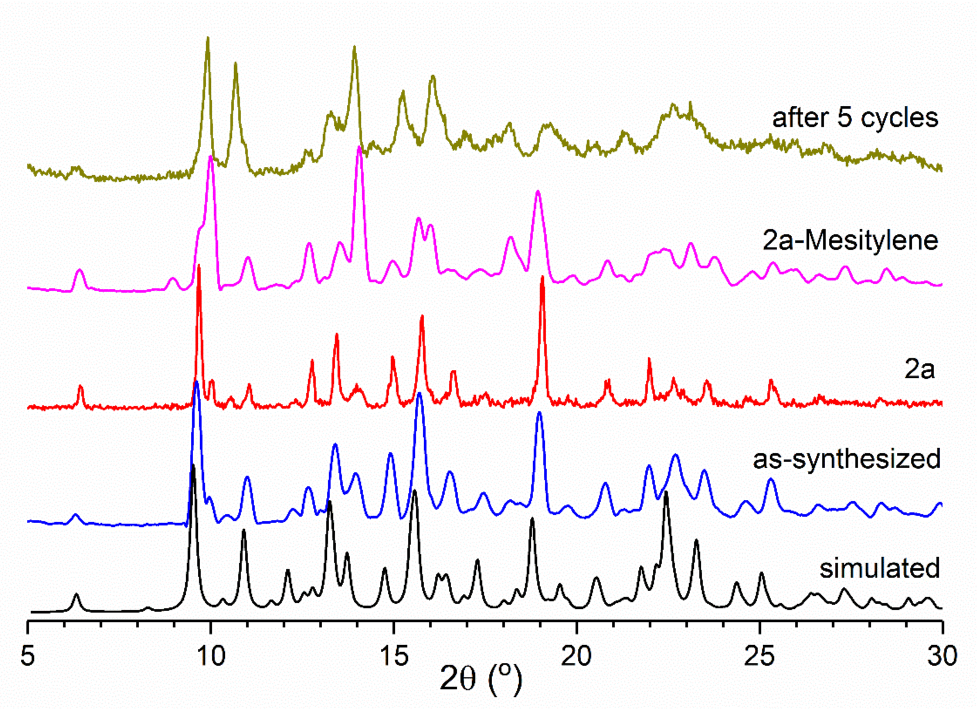

(b).

Figure S16. PXRD patterns for (a) **1a** and (b) **2a** before and after exposure with mesitylene.

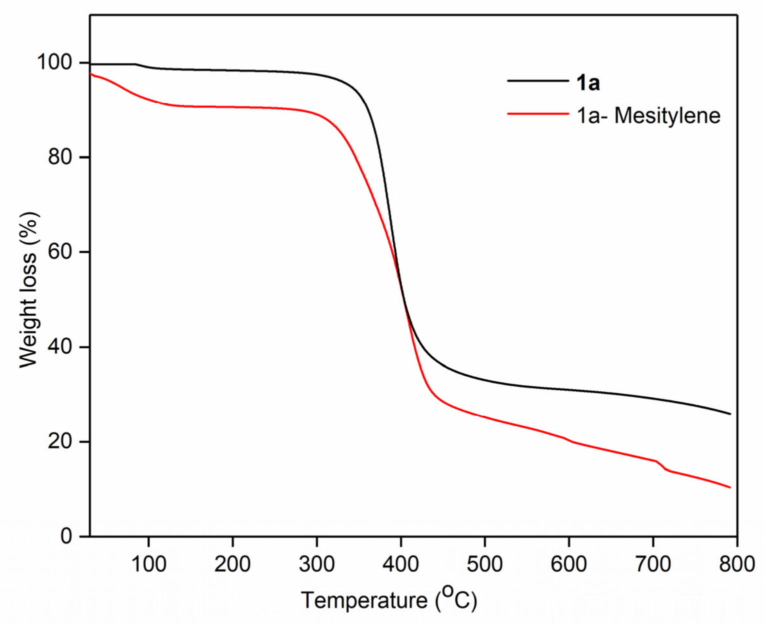

(a).

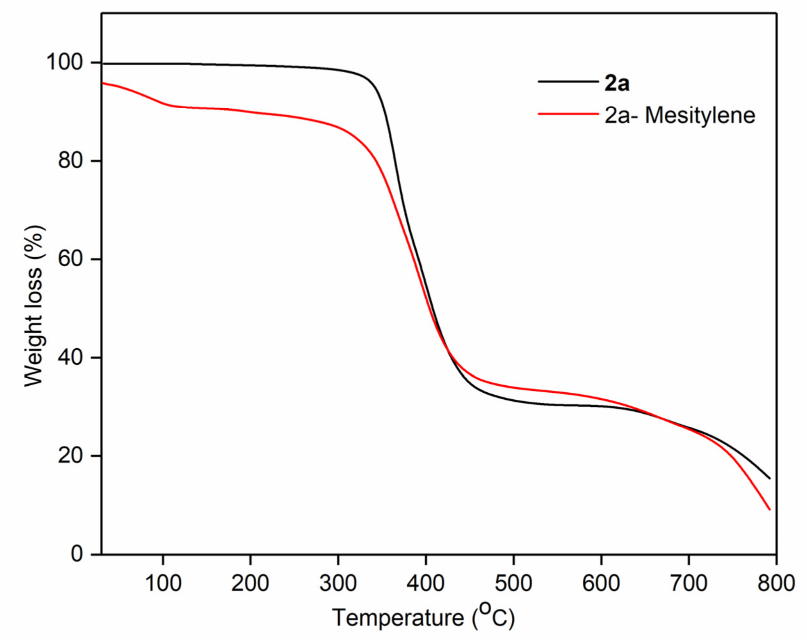

(b).

Figure S17. TGA curves measured for (a) **1a** and mesitylene-loaded **1a** and (b) **2a** and mesitylene-loaded **2a**.

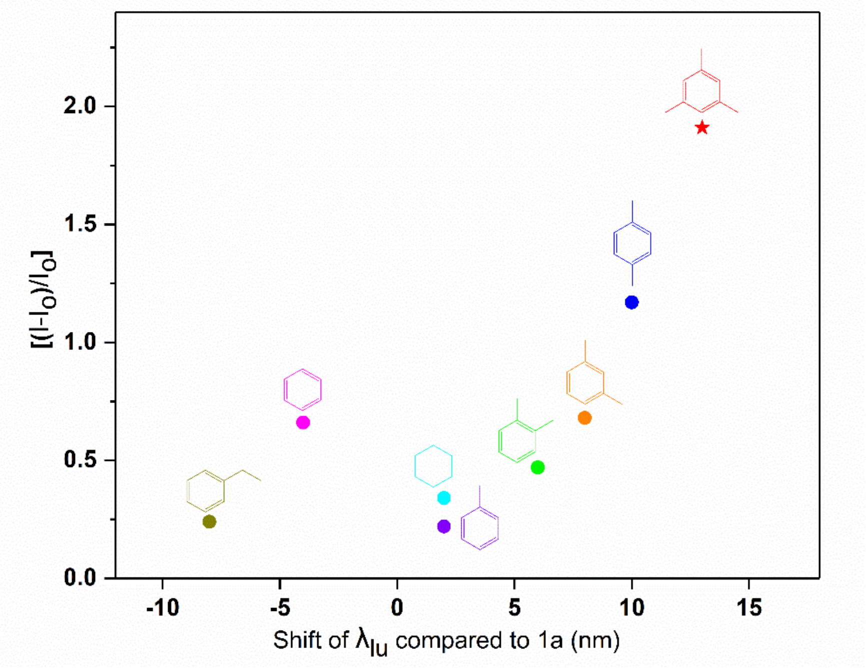

(a).

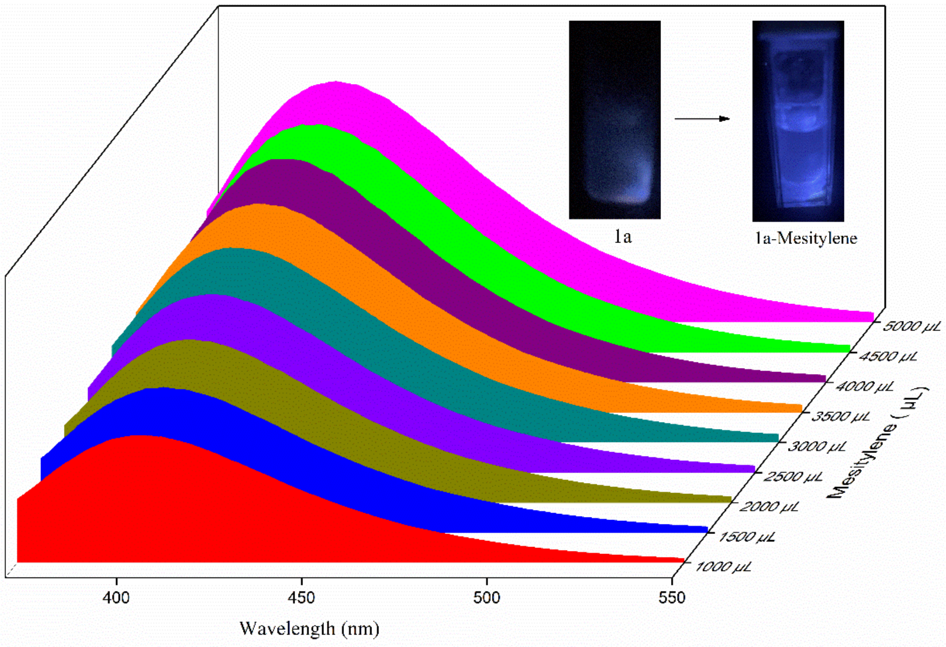

(b).

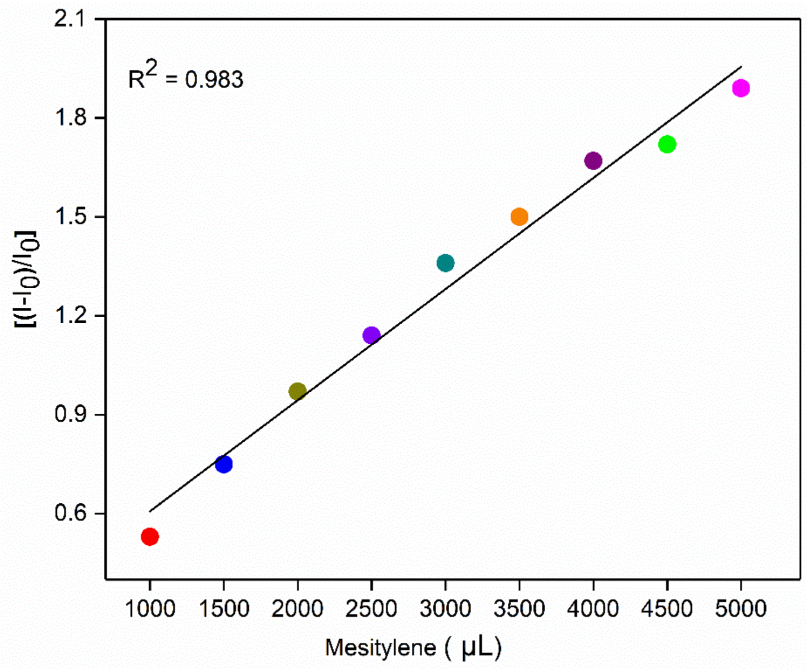

(c).

Figure S18. (a) The relationship of luminescence intensities of VOCs-loaded **1a** versus emission wavelength shifts of λ_{lu} compared to **1a**. (b) Emission spectra of **1a** with increasing amount of mesitylene upon excitation at 350 nm. (c) The calibration curves showing luminescence intensity of **1a** at different amount of mesitylene.

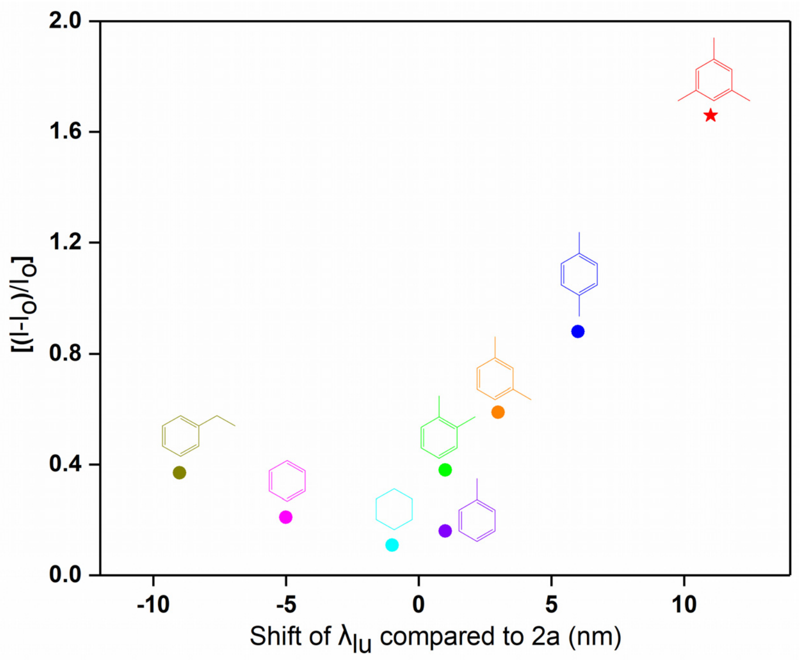
2.0
1.6
1.2
0.8
0.4
0.0
-10
-5
0
5
10
[(I-I0)/I0]
Shift of λlu compared to 2a (nm)

(a).

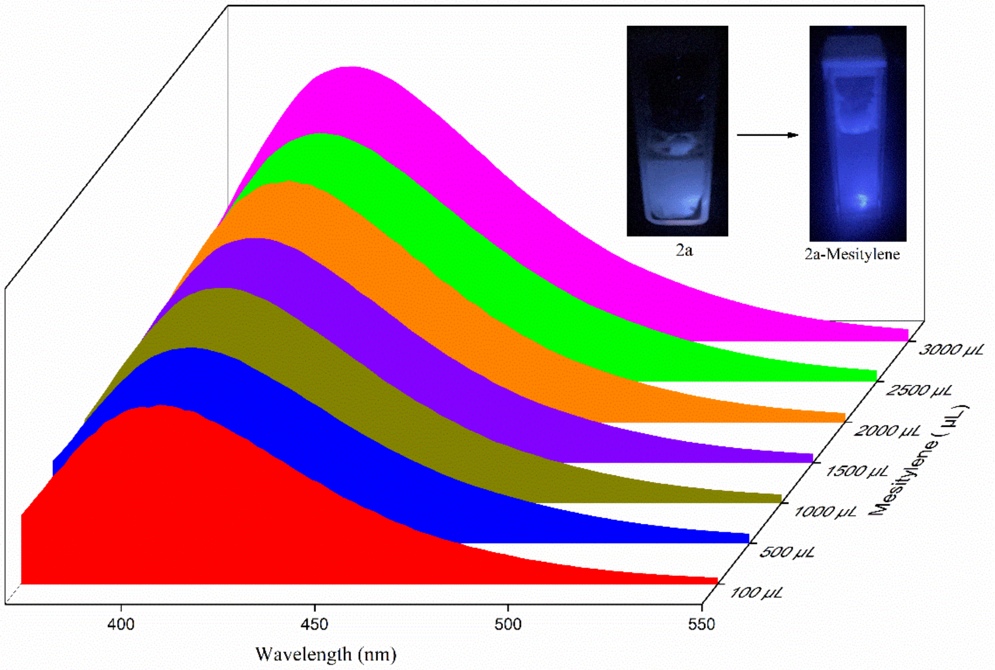
2a
2a-Mesitylene
3000 μL
2500 μL
2000 μL
1500 μL
1000 μL
500 μL
100 μL
Mesitylene (μL)
400
450
500
550
Wavelength (nm)

(b).

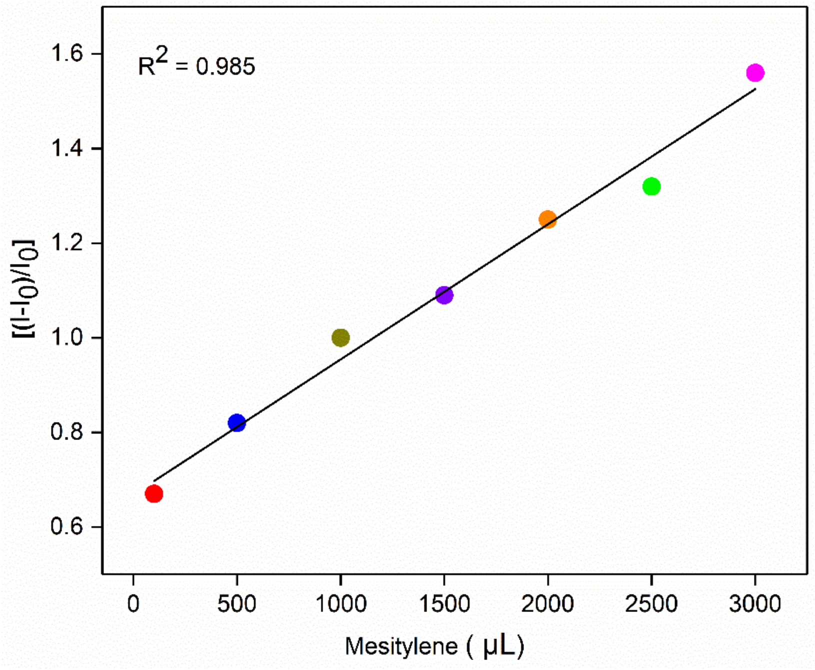

(c).

Figure S19. (a) The relationship of luminescence intensities of VOCs-loaded **2a** versus emission wavelength shifts of λ_{lu} compared to **2a**. (b) Emission spectra of **2a** with increasing amount of mesitylene upon excitation at 350 nm. (c) The calibration curves showing luminescence intensity of **2a** at different amount of mesitylene.

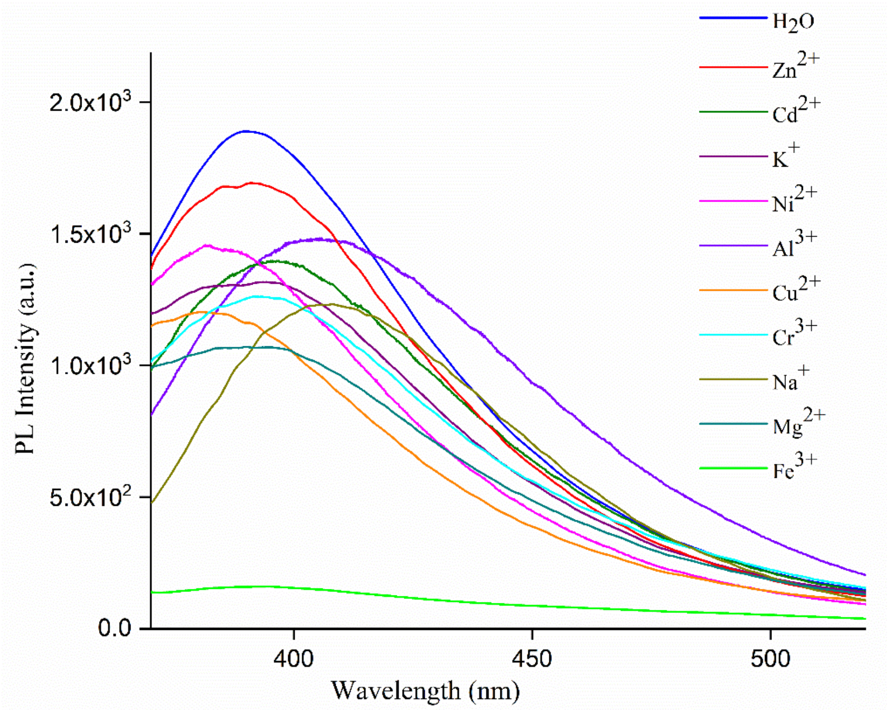

(a).

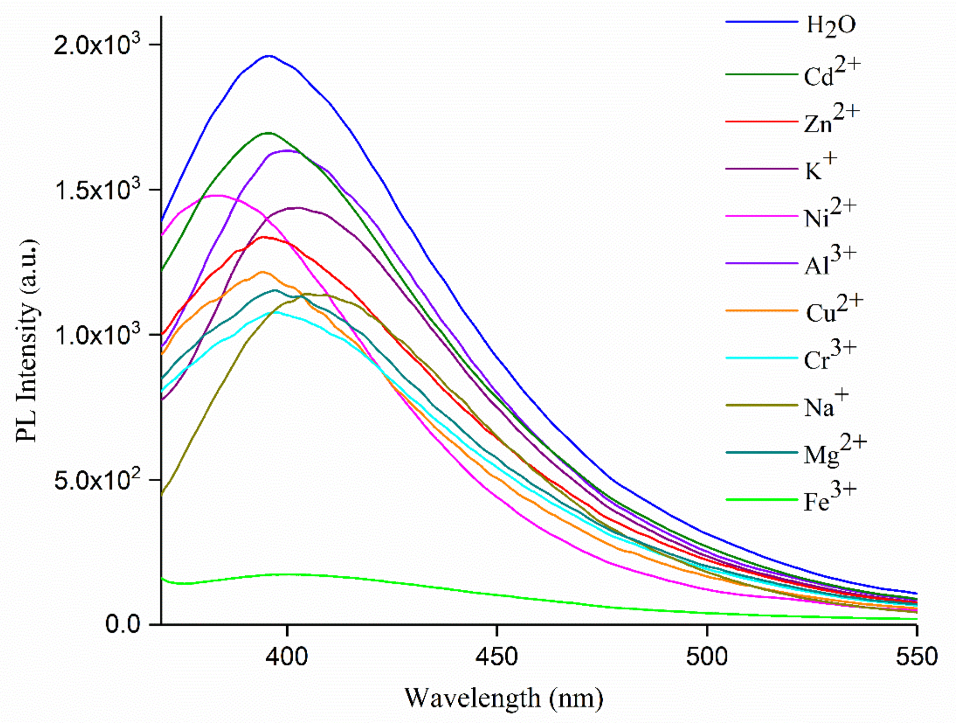

(b).

Figure S20. Luminescence responses towards aqueous solutions of various metal cations upon excitation at 350 nm for (a) **1** and (b) **2**.

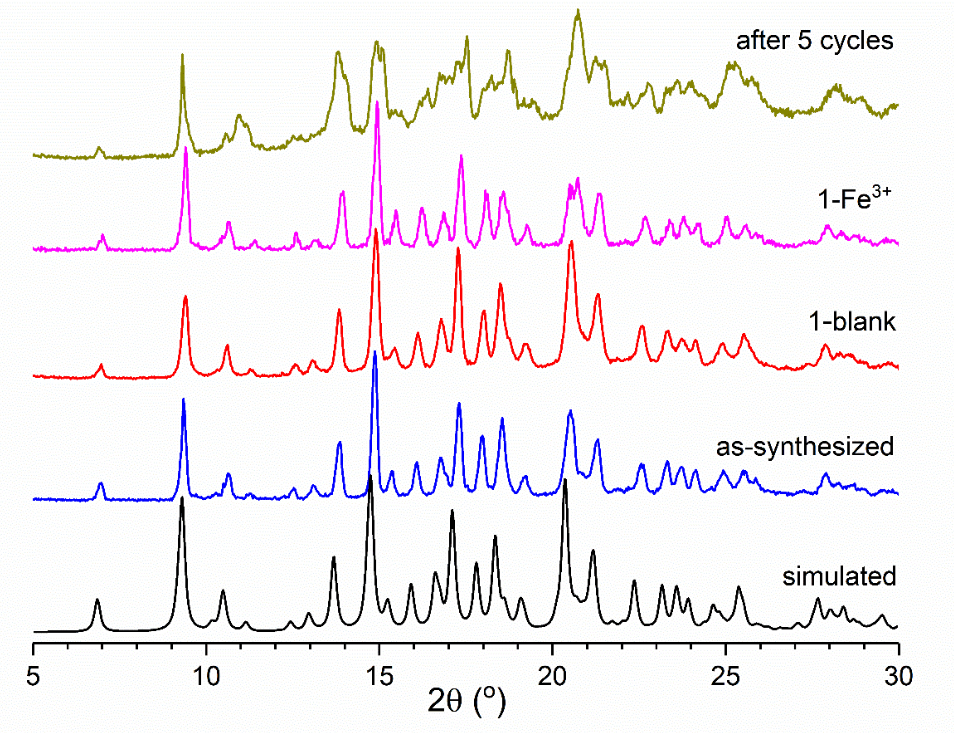

(a).

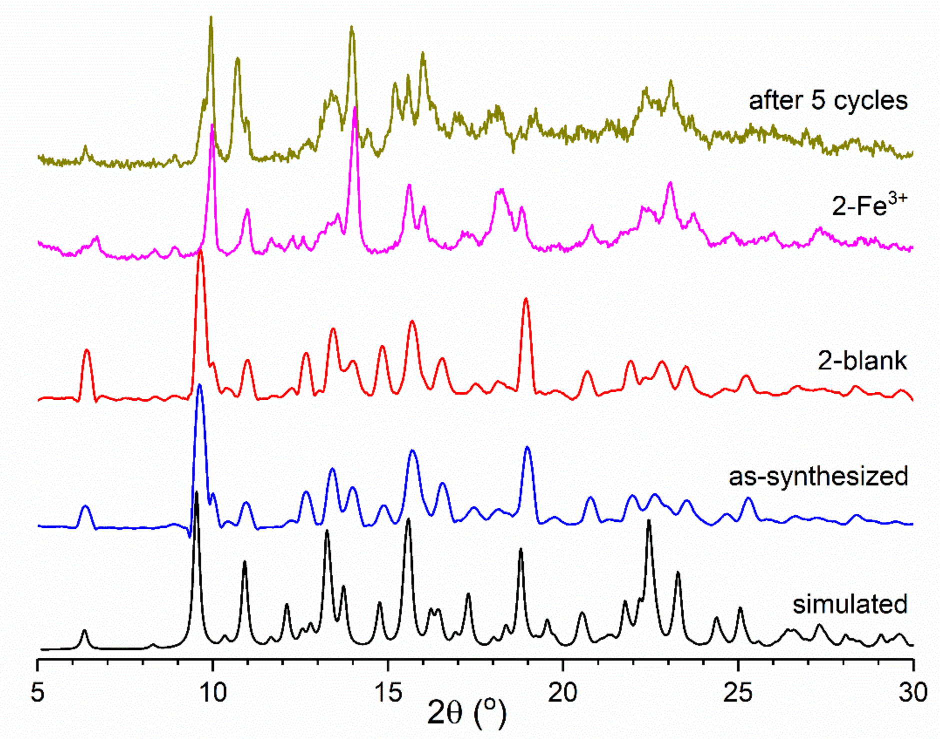

(b).

Figure S21. PXRD patterns before and after treatments with Fe^{3+} ions for (a) **1** and (b) **2**.

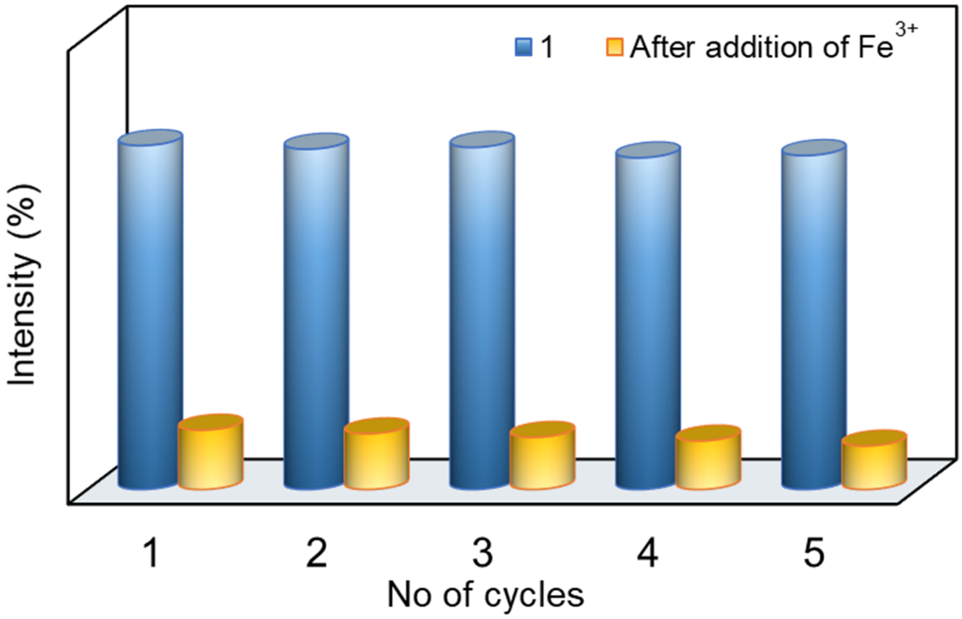

(a).

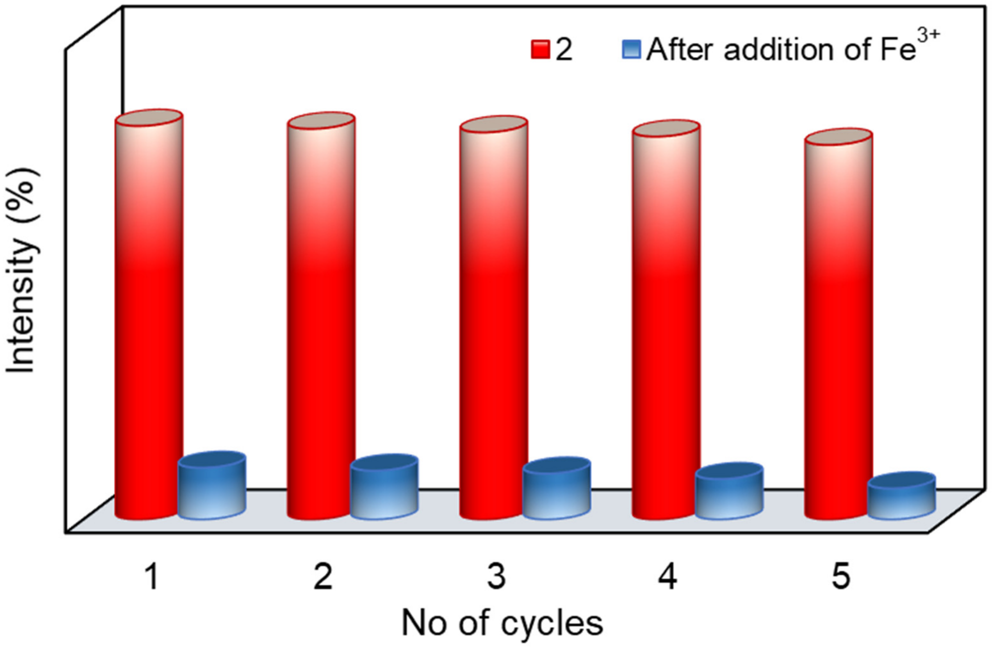

(b).

Figure S22. Bar diagrams showing the emission intensities of (a) **1** and (b) **2** treated with Fe^{3+} for five repeated cycles.

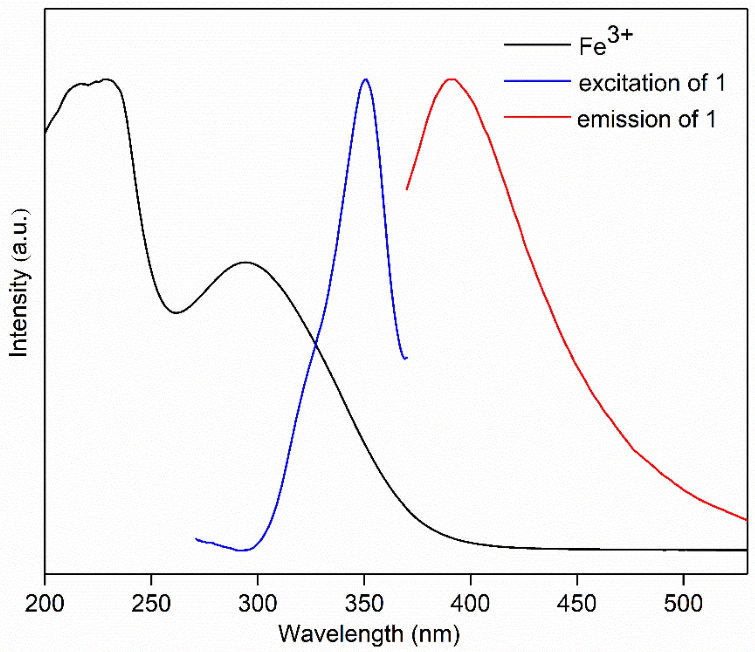

(a).

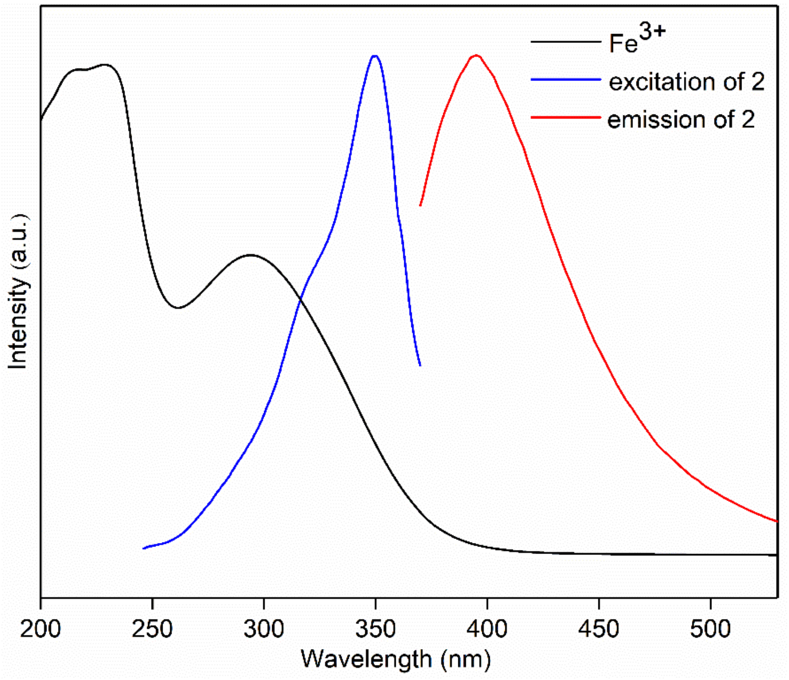

(b).

Figure S23. UV-vis absorption spectrum of Fe^{3+} ions in aqueous solution and the excitation and emission spectra of (a) **1** and (b) **2**.

Table S1. Sensing properties of reported compounds toward various aromatic VOCs.

Compound	Metal	λ_{ex}	λ_{em}	$[(I-I_0)/I_0]$	Analytes	Reference
$[Zn(\mathbf{L})(1,4\text{-}NDC)\cdot H_2O]_n$, **1**	Zn	350	403	1.91	mesitylene	this work.
$[Cd(\mathbf{L})(1,4\text{-}NDC)(H_2O)\cdot MeOH]_n$, **2**	Cd	350	406	1.65	mesitylene	this work
NUS-1a	Zn	400	504		benzene	J. Am. Chem. Soc. **2014**, *136*, 7241.
$Me_2NH_2)[Cd(TTCA)(H_2O)]\cdot 3DMF\cdot H_2O$	Cd	420	501	0.37	m-xylene	Cryst. Growth Des. **2015**, *15*, *7*, 3119.
In-bpy	In	306	476	19.5	p-xylene vapor	Chem. Eur. J. **2018**, *24*, 12474.
$Cd_3(L)(bipy)_2\cdot 4DMA$	Cd	324	427		benzene	**Mater. Horiz. 2015**, **2**, 245-251.
$[Zn_2(\mu_2\text{-}BDC)_2(iQ)_2]$	Zn	330	409	27.2	p-xylene	*Crystals* **2020**, *10*, 5, 344.
NUS-40-Zn	Zn	420	674	2.76	o-xylene	Inorg. Chem. **2018**, *57*, 13631.
$[Cd_2(tppe)(bpdc)_2(H_2O)]$	Cd	380	496	0.90	mesitylene	**Dalton Trans. 2016**, *45*, 14888-14892.

Table S2. Sensing properties of reported compounds toward Fe^{3+}ions.

**Compound**	**Analytes**	**Stern-Volmer constant (K_{sv}, M^{-1})**	**Adjusted R^2**	**Limit of detection (μM)**	**Reference**
$[Zn(L)(1,4\text{-}NDC)\cdot H_2O]_n$, **1**	Fe^{3+}	6.895 x 10^5	0.997	2.35	this work
$[Cd(L)(1,4NDC)H_2O\cdot MeOH]_n$,**2**	Fe^{3+}	9.940 x 10^5	0.975	1.01	this work
$[Zn_2(L)_2(bpe)_2(H_2O)_2]$	Fe^{3+}	2.5 x 10^5		25	**Dalton Trans. 2015**, ***44***, 18795-18803.
$\{[Cd_2(dpc)(bib)(H_2O)]\cdot H_2O\}n$	Fe^{3+}	8.939 x 10^6	0.974		Journal of S. S. Chemistry. **2019**, *277*, 564–574.
$[Bmim][Dy(NO_3)_4]$	Fe^{3+}	2.0×10^6	0.982	9.4	**Dalton Trans. 2016**, ***45***, 1040-1046.
**ZSTU-1**	Fe^{3+}	2.69×10^6	0.990	6.38	Journal of S. S. Chemistry. **2019**, *278*, 120-892.
**$[Cd(L)(4\text{-}CPA)]_n$**	Fe^{3+}	3.866 × 10^5	0.988	4.70	Polyhedron. **2018**, *151*, 530-536.
$\{[Cd_4(HDDCP)_2(4,4'\text{-}bibp)_2(H_2O)_2]\cdot 2.5(DOA)\cdot 1.5(H_2O)\}_n$<br>$\{[Cd_2(HDDCP)(1,4\text{-}bib)(H_2O)]\cdot H_2O\}_n$	Fe^{3+}	2.15 x 10^5<br>8.86 x 10^4	0.998<br>0.995	1.34<br>3.32	**CrystEngComm 2020**, ***22***, 6927.
$\{[Zn(L)(dcdps)]\}n$<br>$\{Zn(L)(bdc)\}n$<br>$\{[Cd(L)(oba)]\cdot 0.5DMF\}_n$<br>$\{[Cd(L)(bdc)\cdot(H_2O)_2]\cdot 2DMF\}_n$	Fe^{3+}	7.004 x 10^3<br>9.066 x 10^3<br>4.984 x 10^3<br>6.387 x 10^3	0.992<br>0.970<br>0.968<br>0.966	6.21<br>4.45<br>11.52<br>6.36	Cryst. Growth Des. **2020**, *20*, 1898.
$[Zn_2(L_{13})_2(bpe)_2(H_2O)_2]_n$	Fe^{3+}	2.39 x 10^3		2.50	Dalton Trans. **2015**, *44*, 18795.
$[Zn(tpb)(Hbtc)]_n$	Fe^{3+}	1.57 x 10^4	0.998	1.95	**Dalton Trans. 2020**, ***49***, 11201.
$\{[Zn(L1)(BTEC)0.5]\cdot 3.1H_2O\}_n$<br>$\{[Zn(L2)0.5(BTEC)0.5]\cdot 1.5H_2O\}_n$<br>$\{[Zn_2(L3)(BTEC)(H_2O)]\cdot H_2O\}_n$<br>$\{[Zn_2(L4)(BTEC)]\cdot H_2O\}_n$	Fe^{3+}	5314<br>6775<br>6636<br>6424	0.994<br>0.990	6.35<br>5.01<br>7.02<br>6.19	**CrystEngComm 2021**, ***23***, 1604.
Eu^{3+}@1	Fe^{3+}	5.12 x 10^3	0.999	0.5	**J. Mater. Chem. A. 2014**, ***2***, 13691.
$\{[Cd(5\text{-}asba)(bimb)]\}_n$	Fe^{3+}	1.78 x 10^4		1.87	**J. Mater. Chem. C. 2016**, ***4***, 11404.
$\{[Zn_2(1,4\text{-}ndc)_2(3\text{-}abpt)]\cdot 2DMF\}_n$<br>$\{[Cd(1,4\text{-}ndc)(3\text{-}abit)]\cdot H_2O\}_n$	Fe^{3+}	2.38 x 10^4<br>9.54 x 10^3	0.992<br>0.993		**New J. Chem. 2017**, ***41***, 8107.
$[Zn_2(oba)_2(bpta)]\cdot 3DMF\}_n$	Fe^{3+}	6.50 x 10^4	0.990	3.00	Polyhedron. **2019**, *166*, 166.
